# Systems biology drug screening identifies statins as enhancers of current therapies in chronic lymphocytic leukemia

**DOI:** 10.1038/s41598-020-78315-0

**Published:** 2020-12-17

**Authors:** Neus Gimenez, Rupal Tripathi, Ariadna Giró, Laia Rosich, Mònica López-Guerra, Irene López-Oreja, Heribert Playa-Albinyana, Fabian Arenas, José Manuel Mas, Patricia Pérez-Galán, Julio Delgado, Elias Campo, Judith Farrés, Dolors Colomer

**Affiliations:** 1 Experimental Therapeutics in Lymphoid Malignancies Group, Institut d’ Investigacions Biomèdiques August Pi i Sunyer (IDIBAPS), Barcelona, Spain; 2 Centro de Investigación Biomédica en Red de Cáncer (CIBERONC), Barcelona, Spain; 3 Anaxomics Biotech, Barcelona, Spain; 4 Hematopathology Unit, Department of Pathology, Hospital Clinic, IDIBAPS, Barcelona, Spain; 5 Centre for Genomic Regulation (CRG), The Barcelona Institute of Science and Technology, Barcelona, Spain; 6 Microenvironment in Lymphoma Pathogenesis and Therapy Group, IDIBAPS, Barcelona, Spain; 7 Department of Hematology, Hospital Clinic, IDIBAPS, Barcelona, Spain; 8 University of Barcelona, Barcelona, Spain

**Keywords:** Cancer, Drug discovery, Systems biology

## Abstract

Chronic lymphocytic leukemia (CLL) is a B lymphoid malignancy highly dependent on the microenvironment. Despite new targeted therapies such as ibrutinib and venetoclax, disease progression and relapse remain an issue. CLL cell interactions with the supportive tissue microenvironment play a critical role in disease pathogenesis. We used a platform for drug discovery based on systems biology and artificial intelligence, to identify drugs targeting key proteins described to have a role in the microenvironment. The selected compounds were screened in CLL cell lines in the presence of stromal cells to mimic the microenvironment and validated the best candidates in primary CLL cells. Our results showed that the commercial drug simvastatin was the most effective and selective out of the tested compounds. Simvastatin decreased CLL cell survival and proliferation as well as cell adhesion. Importantly, this drug enhanced the antitumor effect of venetoclax and ibrutinib. We proposed that systems biology approaches combined with pharmacological screening could help to find new drugs for CLL treatment and to predict new combinations with current therapies. Our results highlight the possibility of repurposing widely used drugs such as statins to target the microenvironment and to improve the efficacy of ibrutinib or venetoclax in CLL cells.

## Introduction

Chronic lymphocytic leukemia (CLL) is a mature B-cell neoplasm characterized by a progressive accumulation of mature functionally incompetent B cell lymphocytes (CD19^+^) in which microenvironmental signals play a critical role in ontogeny and evolution^1^. Recently new targeted therapies have been approved for CLL such as ibrutinib, a BTK inhibitor targeting the B cell receptor (BCR) signaling, and venetoclax, a BCL2 inhibitor. Both drugs are associated with significantly better progression-free survival and overall survival compared to chemoimmunotherapy. However, a subset of patients develops resistance towards these novel drugs and disease relapse^2^.

The microenvironment in the bone marrow and secondary lymphoid organs plays a crucial role in sustaining the viability of CLL cells and still represent a major obstacle to achieve disease eradication^3^. Ibrutinib in addition to interfering with BCR signaling as its primary mechanism of action, appears to block survival signals delivered by the microenvironment, which may include cell–cell contact and cytokines that modulate cell migration, trafficking, and proliferation^4,5^.

Systems biology represents a natural complement to ongoing efforts in cell biology by integrating information about the parts (e.g., genes, proteins) of a complex biological system with the aim to predict the behavior of the whole. Furthermore, it could be a powerful instrument to link pharmacological and disease data, thereby providing a tool to evaluate the pleiotropic effect of large compound libraries and existing drugs^6^. There are many examples of the successful identification of novel therapeutic strategies based on systems biology^7–10^. In this study, bioactive compounds with known protein targets have been screened in silico using a systems biology-based approach^11^ for their potential to target CLL microenvironment. A molecular description of the microenvironment effects in CLL has been extracted from the scientific literature and used as desired targets. The drug screening algorithm identified compounds that where affecting the target area and close neighbors. A selection of the best candidates was tested in vitro in primary CLL cells and CLL cell lines in the absence or presence of the human bone-marrow derived stromal cell line HS-5 to mimic the microenvironment.

## Results

**Figure 1:**
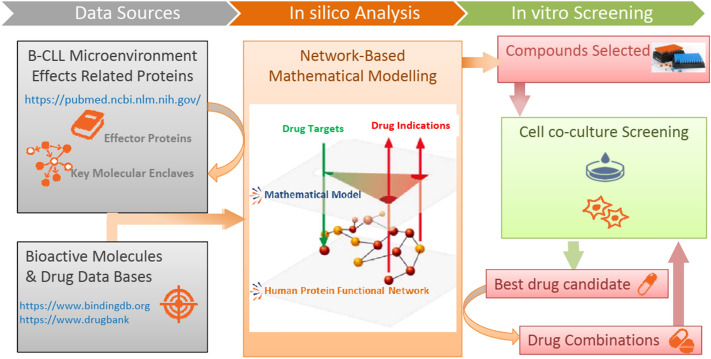
Overall experimental procedure: (1) *Data sources*: Molecular description of CLL microenvironment effects retrieved from literature and mapping in a human protein functional network. Bioactive compounds and drugs with known protein target profile. (2) In silico analysis: Different metrics derived from network-based mathematical models have been used to select key molecular enclaves in the network around the known effector proteins of CLL microenvironment motive; to identify bioactive compounds with a potential effect around the key proteins and to identify antineoplastic agents that could have synergistic effect with simvastatin and (3) In vitro screening. Compound library and combination therapies with simvastatin were screened using an in vitro coculture CLL system.

Based on the existing knowledge on CLL microenvironment and using systems biology approaches, we have identified in silico compounds that potentially target microenvironment signaling pathways. Then, we screened and validated some of these compounds in CLL cells using in vitro assays. The analytical workflow consisted of three interconnected parts: data sources, in silico analysis and in vitro screening are summarized in Fig. 1.

### Identification of key molecular enclaves involved in CLL microenvironment

**Figure 2:**
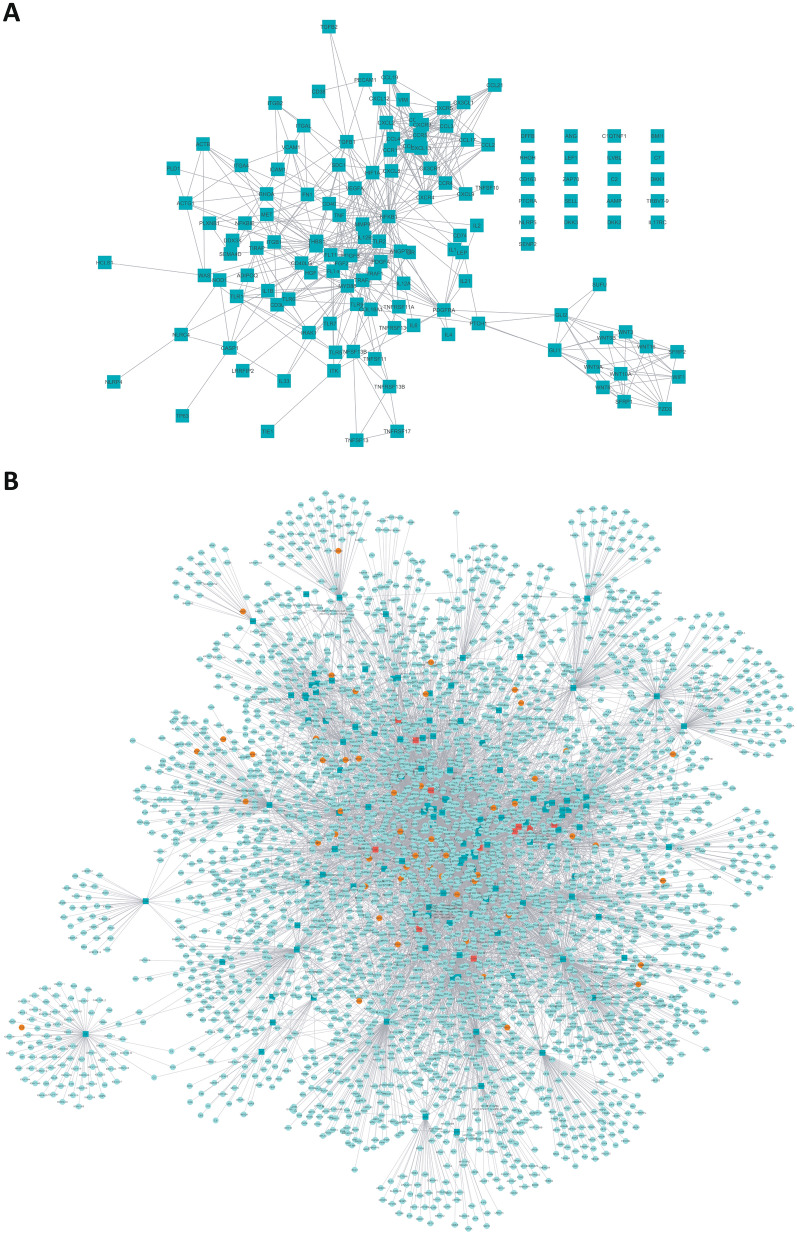
Molecular description of CLL microenvironment and targets. (**A**) 139 proteins (effectors) selected as descriptors of the CLL microenvironment and the relations between them. (**B**) Extract of the network build around the selected effectors of the microenvironment that contains the targets for the 65 screened compounds. Dark green square: CLL microenvironment selected descriptors; Light green round: Proteins directly related with selected CLL microenvironment descriptors; Orange, round: Protein targets of the 65 screened compounds that lay within the network described by the microenvironment effector proteins and close neighbours; Red square: Proteins that are effectors and targets simultaneously. The images haven been developed with Cytoscape v3.7.2 software^51^.

A molecular description of CLL microenvironment was obtained by the identification of effector proteins with a known role on CLL microenvironment as described in methods section. In Supplemental Table S1 are listed the 139 proteins and the references (PMID) used to select the proteins. The causative effect denotes the cause of the pathological behavior according to the literature, being 1 if the protein is more active or − 1 if it is more inactive. The known functional associations of these proteins with other proteins where retrieved from public data bases and we built a network around the known proteins related with CLL microenvironment effect. The contributions of close neighbor proteins on the effector proteins were also taken into consideration. To narrow down the target area of analysis a combination of different systems biology measures based on the human protein functional microenvironment network was used. Firstly, we used the artificial neural networks (ANN)^11^ based model to identify effectors that connect with most of the other effectors. Proteins that are closer to a higher number of other proteins in the microenvironment motive have more chances to be a good target. Secondly, we used a mathematical modeling strategy based on sampling methods^12^ to select those with higher impact on the whole response when their action in disease stage was reverted. The combination of both analyses has identified a subset of 57 proteins, from now on referred as “key proteins” (Fig. 2A and Supplemental Table S2).

### Compound library selection

The same network-based mathematical model of ANN^11^ was used to select compounds with the best target combination to affect the larger number of key proteins or effectors of the CLL microenvironment. Compounds were selected from BindingDB and DrugBank databases based on three criteria: (i) compounds with a predictive score > 78 and a p-value lower than 0.05 using the ANN algorithm^11^, (ii) linkage to a drug supplier and (iii) among the compounds fulfilling the previous criteria with the same target profile, the one with the best reported binding constant was selected. Finally, the number of compounds moving forward to the phenotypic screening was 65 (54 bioactive compounds and 11 drugs) (Supplemental Table S3). The number of targets affected by the selected compounds is represented in Fig. 2B.

### Compound library screening in CLL cells

**Figure 3:**
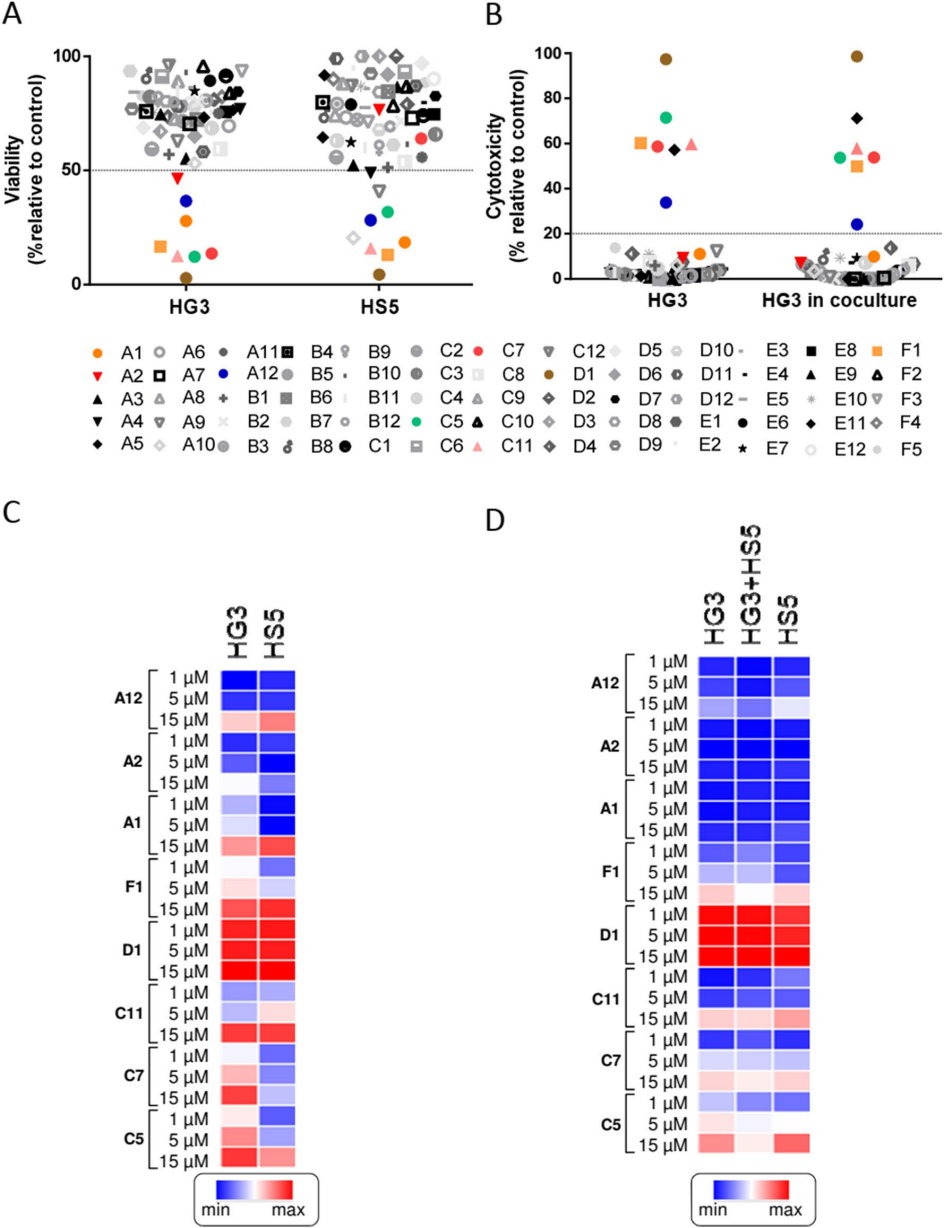
Compound library screening in HG3 and HS-5 cell lines. (**A**) Viability of cells treated with the compounds at the concentration of 15 µM in the HG3 and HS-5 cell lines. (**B**) Cytotoxicity of the compounds at 15 µM concentration in HG3 alone and in coculture with HS-5. (**C**) Heat maps showing viability of cells treated with the selected compounds (A12, A2, A1, F1, D1, C11, C7,C6) at the concentrations of 1–5–15 µM in the HG3 and HS-5 cell lines. (**D**) Heat maps showing cytotoxicity of the selected compounds (A12, A2, A1, F1, D1, C11, C7,C6) at the doses of 1–5–15 µM in HG3, HG3 in coculture with HS-5 and in HS-5. Cells were treated for 48 h with the compounds at concentrations from 1 to 15 µM. Each dot (**A**,**B**) and each box (**C**,**D**) represent the mean of 3 independent experiments. Dotted line in (**A**,**B**) indicates the threshold to discriminate the effect of the compounds. The cut off for MTT and cytotoxicity was 50% and 20%, respectively. Viability of CLL cells was measured using the MTT assay, and is depicted relative to untreated control. Cytotoxicity was defined as the increase in Annexin-V+/PI + cells compared to untreated control.

The CLL HG3 cell line and the human bone marrow stromal HS-5 cell line were used to test the effect of the 65 selected compounds. MTT analysis was performed after incubation of cells for 48 h with 15 µM of the different compounds (Fig. 3A). Only 8 compounds were affecting cell viability (color dot) taking into account a threshold of 50% determined by a ROC analysis (Supplemental Figure S1A). Cytotoxicity was also analyzed by Annexin-V/PI staining in HG3 alone or in coculture with the stromal cell line HS-5 (Fig. 3B). Six compounds were cytotoxic for HG3 cells using a threshold of 20% determined by a ROC analysis (Supplemental Figure S1B). One of these compounds (A5) was a false positive due to autofluorescence. Similar results were obtained when HG3 cells were cocultured with HS-5 cells (Fig. 3B). Two compounds (A1 and A2) exerted a cytostatic effect (MTT analysis) but with no effect on cell death (Annexin V/PI staining). These results were also confirmed in the MEC-1 CLL cell line (Supplemental Figure S2). Then, a dose response (1 to 15 µM) was performed in the HG3 and HS-5 cell lines with the 8 selected compounds. At all concentrations used, the CLL cell line was more sensitive to these compounds in a dose dependent manner by MTT (Fig. 3C) or Annexin-V analysis (Fig. 3D) than the stromal HS-5 cell line. Compound D1 exerted a high cytotoxic effect in all cell lines even at the lowest doses used.

### Compound library screening in primary CLL cells

**Figure 4:**
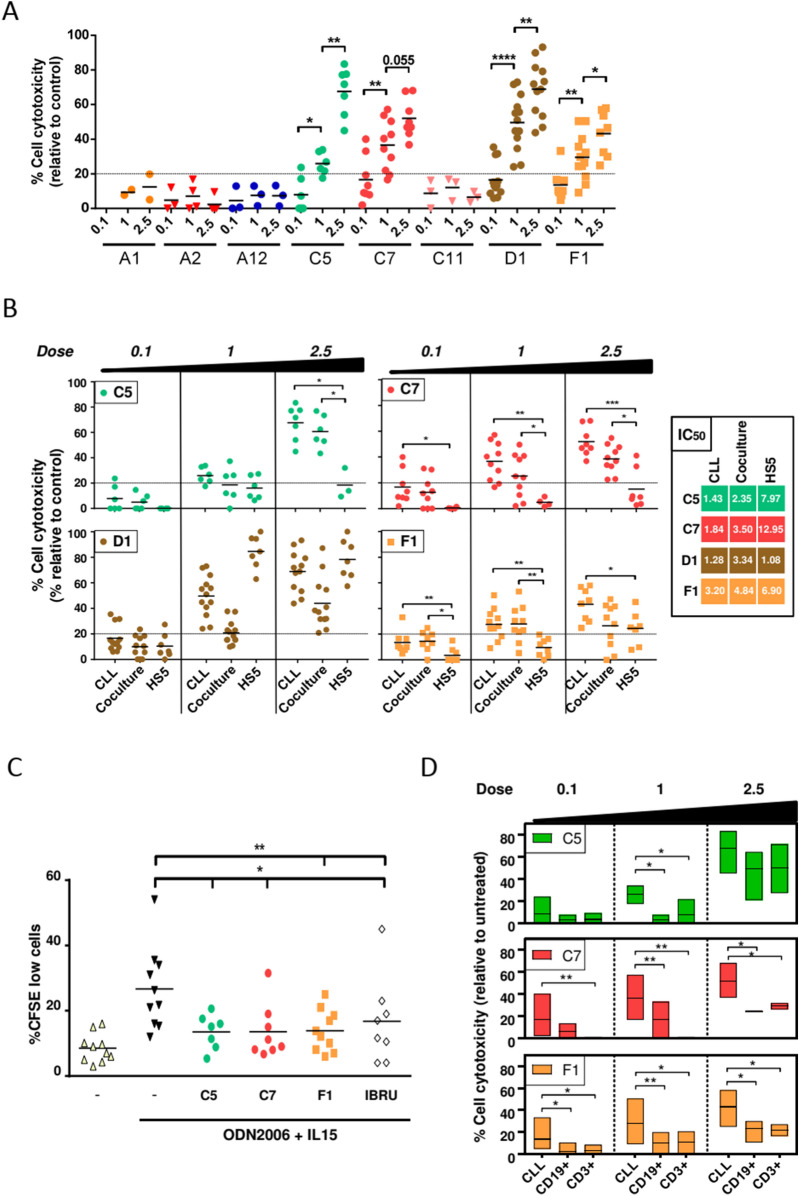
Screening of selected compounds in primary CLL cells. (**A**) Cytotoxicity of the compounds at 0.1–1–2.5 µM in primary CLL cells (n = 2–13). CLL cells were treated for 48 h with the compounds at concentrations from 0.1 to 2.5 µM. Dotted line indicates the threshold to discriminate the effect of the compounds. The cut off for cytotoxicity was 20%. Cytotoxicity was defined as the increase in Annexin-V^+^/PI^+^ cells compared to untreated control. (**B**) Cytotoxicity of the compounds at 0.1–1–2.5 µM concentration in primary CLL cells, primary CLL in coculture with HS-5 cells and HS-5 cells alone (n = 6–13). CLL cells were treated for 48 h with the compounds at concentrations from 0.1 to 2.5 µM. Dotted line indicates the threshold to discriminate the effect of the compounds. The cut off for cytotoxicity was 20%. Cytotoxicity was defined as the increase in Annexin-V + /PI + cells compared to untreated control. The IC50 has been calculated and presented for each condition. (**C**) Percentage of proliferating CD19^+^ CLL cells after ODN2006 + IL15 stimulation (30 min before treatment) and treatment with different compounds at the dose of 1 µM (C5, C7 and F1) or 0.25 µM (Ibrutinib) for 6 days measured by CFSE dilution (n = 10). (**D**) Cytotoxicity of the compounds at 0.1–1–2.5 µM concentration in primary CLL cells and in CD19 and CD3 positive cells from healthy donors (n = 8) after 48 h of incubation. Line into the bars represents the mean of all samples analyzed. Non-parametric Wilcoxon matched-pairs signed rank test was used for statistical analysis. *P < 0.05, **P < 0.01, ***P < 0.001, ****P < 0.0001.

A dose–response screening of these 8 selected compounds was performed in primary CLL cells with doses ranging from 0.1 to 2.5 µM. Compounds C5, C7, D1 and F1 exerted a significant dose-dependent cytotoxic effect (Fig. 4A). Compounds A1, A2, A12 and C11 did not exert any cytotoxic effect regardless of the doses used. Then, these 4 active compounds (C5, C7, D1 and F1) were analyzed in primary CLL cells in the presence of HS-5 in order to mimic the microenvironment. Compound D1 was discarded for not being selective for tumoral CLL cells, as a high cytotoxic effect was observed on HS-5 cells. Compounds C7 and F1 were selective for CLL cells even in the presence of HS-5 cells at all tested doses. In contrast, compound C5 only exerted a significant (p < 0.05) and selective effect at the dose of 2.5 µM (Fig. 4B). The IC50 for each compound in the different conditions tested are showed in Fig. 4B.

We also analyzed if these compounds had any effect on CLL proliferation. CFSE-labeled primary CLL cells were induced to proliferate by incubating them with a medium containing the CpG oligonucleotide ODN2006, which triggers growth and cell division in the proliferative centers of CLL patients, and the inflammation-linked cytokine IL-15, which is constitutively produced by stromal cells^13^ for 6 days. As shown in Fig. 4C, ODN2006 plus IL15 induced an increase of CFSE^low^ viable CLL cells indicative of increased cell proliferation. All compounds tested (C5, C7 and F1) decreased the percentage of CFSE^low^ viable CLL cells, indicating that these compounds induced a significant reduction on CLL proliferation. Ibrutinib 0.25 µM was used as a positive control to inhibit proliferation of CLL cells under these conditions. In order to analyze if the cytotoxicity effect of these compounds was specific for CLL cells, we incubated PBMCs from healthy donors with these compounds at the same doses used in primary CLL cells. The effect on normal B (CD19^+^) and T (CD3^+^) lymphocytes was analyzed by flow cytometry. Compounds C7 and F1 were selective for CLL cells at all doses used. In contrast, compound C5 lost the selectivity at 2.5 µM, the highest dose tested (Fig. 4D).

### Target validation of selected compounds

**Figure 5:**
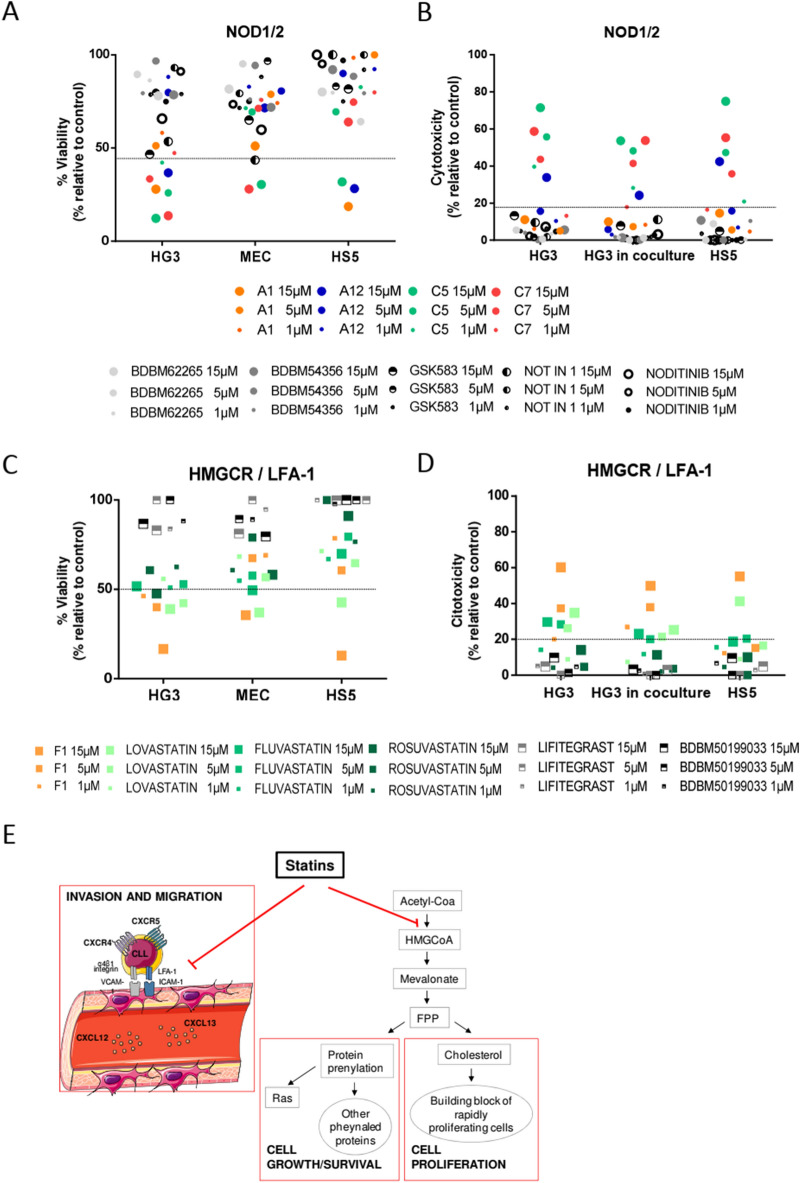
Validation of selected targets. Cells were treated for 48 h with the compounds at concentrations from 1 to 15 µM. Each dot represents the mean of 3 independent experiments. Dotted line indicates the threshold to discriminate the effect of the compounds. The cut off for MTT and cytotoxicity was 50% and 20% respectively. Viability of CLL cells was measured using the MTT assay and is depicted relative to untreated control. Cytotoxicity was defined as the increase in Annexin-V +/PI+  cells compared to untreated control. (**A**) Viability of cells treated with A1, A12, C5 and C7 and NOD1/2 specific inhibitors in HG3, MEC1 and HS-5 cell lines. (**B**) Cytotoxicity of A1, A12, C5 and C7 and NOD1/2 specific inhibitors in HG3, HG3 in coculture with HS-5 and in HS-5 cells. (**C**) Viability of cells treated with the F1 compound (simvastatin) and different statins and specific LFA-1 inhibitors in the HG3, MEC and HS-5 cell lines. (**D**) Cytotoxicity of F1 compound and different statins and specific LFA-1 inhibitors in HG3 alone, HG3 in coculture with HS-5 and in HS-5 alone. Round dots: compounds that target NOD1/2; Square dots: compounds that target HMGCR and LFA-1; Grey dots: specific inhibitors for each target. (**E**) Schematic description of statins mechanism of action.

**Figure 6:**
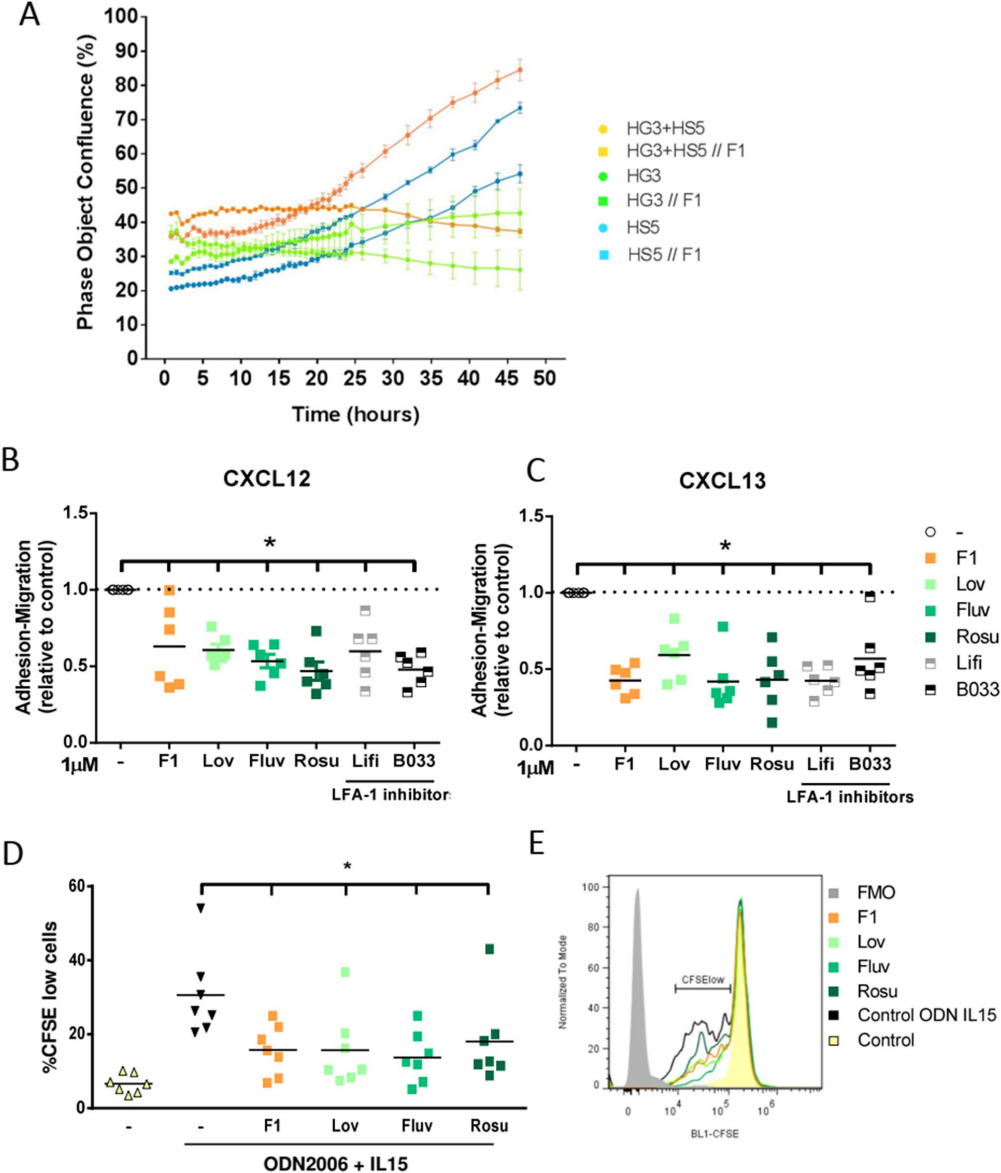
Statins reduce CLL migration, proliferation and viability. Cells were treated for 48 h with the compounds at concentrations of 1 µM. (**A**) Cell phase object confluence after F1 treatment which was monitored for 48 h by recording phase images using the IncuCyte Live Cell Analysis Imaging System in HG3, HS-5 and coculture of HG3 and HS5 cells (n = 3). Bars represent the ± SD of all samples analyzed. (**B**) CXCL12-induced adhesion/migration of CLL cells treated with statins or LFA-1 inhibitors for 3 h and analyzed by transwell assays. Values are presented as the ratio of migrating cells and total viable cells, relative to the untreated control. Dotted line indicates the untreated control reference. (**C**) CXCL13-induced adhesion/migration of CLL cells treated with statins or LFA-1 inhibitors for 3 h and analyzed by transwell assays. Values are presented as the ratio of migrating cells and total viable cells, relative to the untreated control. Dotted line indicates the untreated control reference. (**D**) Percentage of proliferating CD19^+^ CLL cells after ODN2006 + IL15 (ODN IL15) stimulation (30 min before treatment) and treatment with different drugs for 6 days measured by CFSE dilution (n = 7). (**E**) Flow cytometry histograms of a CLL representative case (#4) show the percentages of proliferating cells (gated on viable CD19+ cells) after 6 days of ODN2006 + IL15 stimulation. A decrease in CFSE signal is indicative of cells that have divided. FMO, fluorescence-minus-one. Non-parametric Wilcoxon matched-pairs signed rank test was used for statistical analysis. *P < 0.05. Lov: lovastatin, Fluv: fluvastatin, Rosu: rosuvastatin, Lifi: lifitegrast and B033: BDBM50199033B033.

**Figure 7:**
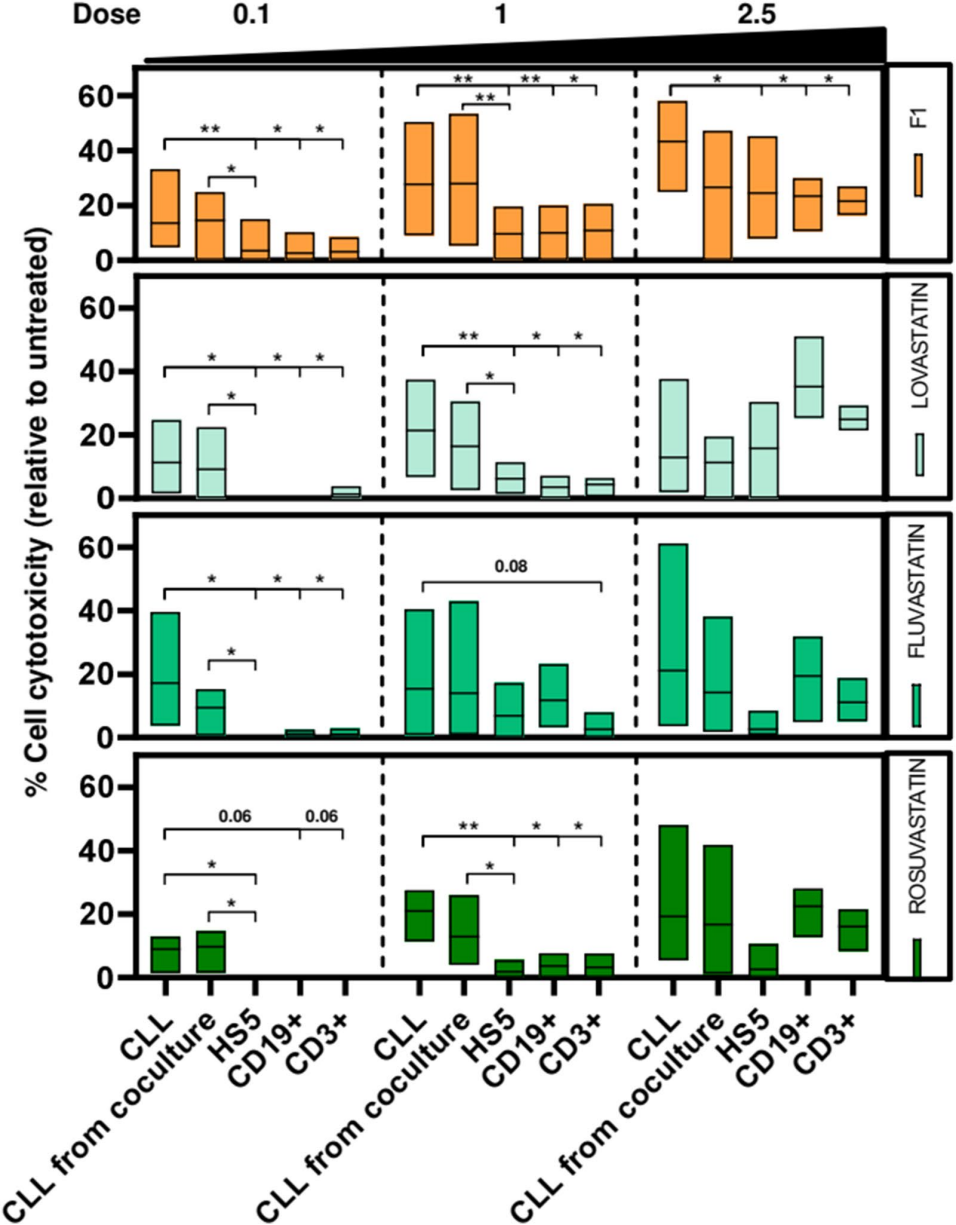
Statins exert a cytotoxic effect in primary CLL cells alone or in coculture with stromal cells. CLL cells or PBMCs from healthy donors were treated for 48 h with the statins at concentrations from 0.1 to 2.5 µM. Cytotoxicity was defined as the increase in Annexin-V^+^/PI^+^ cells compared to untreated control. Cytotoxicity of statins in primary CLL cells alone, in coculture with HS-5 or in HS-5 alone (n = 6) and CD19^+^ B cells and CD3^+^ T cells from healthy donors (n = 8). Line into the bars represents the mean of all samples analyzed. Non-parametric Wilcoxon matched-pairs signed rank test was used for statistical analysis. *P < 0.05, **P < 0.01.

Two compounds were selected C7 and F1. One of the main targets of compound C7 was NOD1 (nucleotide-binding oligomerization domain-containing protein 1) (Supplemental Table S4). NOD1 is an innate immune receptor which together with NOD2 recognizes intra-cellular bacterial components^14^. As NOD1 was also a target of other possible effective compounds (A1, A12 and C5) we compared the cytotoxic effect of these compounds with 5 currently available commercial NOD inhibitors (Supplemental Table S5): BDBM62265, BDBM54356, NOD-IN-1 and noditinib (NOD1 inhibitors) and GSK583 (inhibitor of RIP2, a downstream effector of NOD1/2)^15^. Any of these specific inhibitors exerted any effect on the viability of CLL cell lines (HG3, MEC-1) and the stromal HS-5 cell line analyzed by MTT analysis (Fig. 5A). We confirmed by AnnexinV/PI staining that these inhibitors were not cytotoxic for HG3 alone or in coculture with HS-5 cells (Fig. 5B). Therefore, we considered that NOD1 was not the main target responsible of the effects seen with C7, although we cannot disregard that NOD1 had some pleiotropic effects with other identified targets for this compound. F1 that corresponded to simvastatin was the other compound of interest. (Supplemental Table S3). This drug inhibits the synthesis of cholesterol in the liver by the enzyme 3-hydroxy-3-methylglutaryl coenzyme A reductase (HMGCR)^16^. We compared the effect of simvastatin (F1) with other commercially available stains (lovastatin, fluvastatin and rosuvastatin) with different IC50 (Supplemental Table S5 and Supplemental Figure S3). As it has been reported that statins also inhibit the integrin LFA-1^17^, we also tested two specific LFA-1 inhibitors (lifitegrast and BDBM50199033). We observed that all statins exerted a cytotoxic effect, analyzed by MTT (Fig. 5C) and AnnexinV/PI staining (Fig. 5D), although simvastatin (F1) was the statin with the highest effect. In contrast, LFA-1 inhibitors did not show any cytotoxic effect. Again, we cannot rule out a possible contribution of LFA-1 when combined with HMG-CoA reductase in the effects seen. In this way, we hypothesized that statins through the inhibition cholesterol synthesis pathway, might participate in the decrease of cell survival and proliferation and in addition, they might also influence cellular adhesion by inhibiting LFA-1 (Fig. 5E). To validate this hypothesis, first we analyzed the confluence of cell culture of HG3 and HS-5 alone and HG3 in coculture with HS-5 treated with simvastatin 1 µM for 48 h. We observed that simvastatin induced a dramatic decrease on cell proliferation in HG3 both in the absence and in the presence of HS-5 cells. In contrast, no effect was observed in HS-5 alone (Fig. 6A). We next analyzed the effect of statins and LFA-1 inhibitors on ICAM-mediated adhesion and migration of CLL cells triggered by CXCL12 and CXCL13, key chemokines for CLL cell homing to lymphoid tissues^3^. All different statins and specific LFA-1 inhibitors induced a significant reduction (p < 0.05) on CLL adhesion/invasion induced by CXCL12 (Fig. 6B) and CXCL13 (Fig. 6C). We also confirmed by CFSE staining that all statins tested reduced significantly (p < 0.05) the proliferation of CLL cells induced by incubation of cells with ODN2006 plus IL15 at 6 days (Fig. 6D). Stains decreased the percentage of CFSE^low^ CLL cells as shown in Fig. 6E. To further study the effect of statins in CLL cells, we incubated primary CLL cells alone or in coculture with the stromal cell line HS-5 with these different drugs. As observed in Fig. 7, all statins tested induced a cytotoxic effect on CLL cells, and this effect was not protected by incubating the cells with the stromal cell line HS-5. Furthermore, this effect is selective for CLL cells, as no effect was observed in the HS-5 cells at the low doses tested (0.1 and 1 µM; Fig. 7). The effect of these statins was also analyzed in PBMCs from healthy donors (Fig. 7). Statins exerted a significant selective cytotoxic effect in CLL cells compared to B (CD19^+^) and T (CD3^+^) cells from healthy donors at the doses of 0.1 and 1 µM.

### Systems biology analysis of possible combination therapies

To identify if statins could improve CLL current treatments, we looked for the best combination therapies with simvastatin using the same network-based mathematical model screening used for single drug screening^11^. Drugs were selected when the prediction score for the combination was superior to the individual ones and the p-value associated with the prediction score was lower than 0.1. As simvastatin displayed a high prediction score for CLL (75), it was difficult to identify compounds that increased its individual action. We tested 22 drugs commonly used for CLL treatment and 5 of them showed a probability for the combination above threshold, but only for ibrutinib and venetoclax the combination score was higher than the individual ones (Supplemental Table S6).

### Validation in vitro of combination therapies with statins

**Figure 8:**
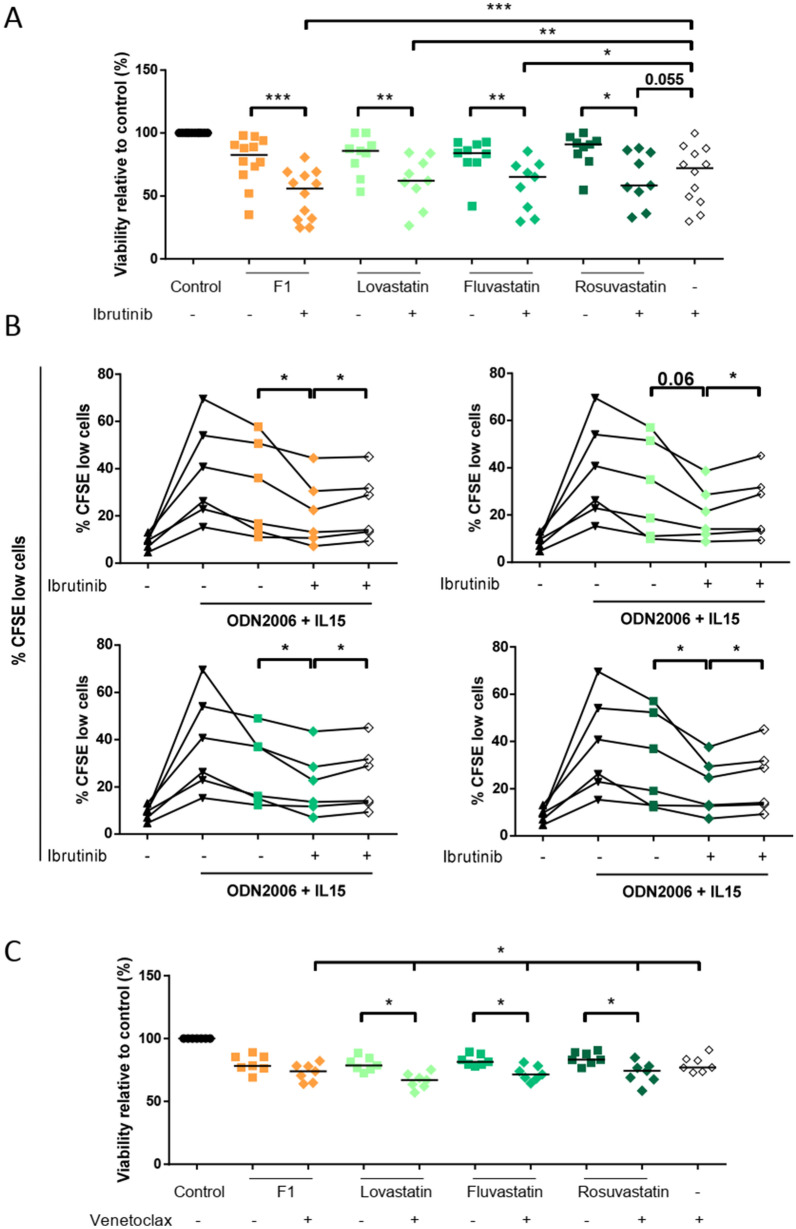
Combinatory effects of statins with venetoclax or ibrutinib. Primary CLL cells were treated with 0.1 µM statins, 1 nM venetoclax or 0.1 µM ibrutinib. (**A**) Viability of primary CD19^+^ CLL cells after incubation of cells with statins and ibrutinib for 6 days (n = 9–12). Percentage of viable cells was measured as CD19^+^Annexin-V^−^ cells by flow cytometry and normalized to untreated control samples. (**B**) Percentage of proliferating CD19^+^ CLL cells after ODN2006 + IL15 stimulation (30 min before treatment) and treatment with statins and ibrutinib for 6 days measured by CFSE dilution (n = 6). (**C**) Viability of CD19^+^ CLL cells after incubation with statins and venetoclax for 48 h (n = 7). Non-parametric Wilcoxon matched-pairs signed rank test was used for statistical analysis. *P < 0.05, **P < 0.01, ***P < 0.001.

According to the systems biology prediction, we tested the combination of statins with ibrutinib and venetoclax. We incubated CLL cells in the presence of ODN2006 plus IL15 for 6 days, and CLL cell-viability was analyzed in CD19^+^ CLL cells by AnnexinV staining. We observed that incubation of cells with different statins and ibrutinib (0.1 µM) reduced CLL cell viability significantly (Fig. 8A). Furthermore, proliferation of CLL cells decreased after ibrutinib and statins when were used alone and this effect on CLL proliferation was significantly higher when statins were combined with Ibrutinib (Fig. 8B). Also, the cytotoxic effect of venetoclax 1 nM increased with the addition of statins 0.1 µM. We observed a significant (p < 0.05) decrease in cell viability when venetoclax and statins were incubated together (Fig. 8C).

## Discussion

CLL is a malignancy of antigen-experienced mature B lymphocytes, in which microenvironmental signals play a critical role in ontogeny and evolution^18^. Recently, ibrutinib^19,20^, a first-in-class Bruton’s tyrosine kinase (BTK) inhibitor and venetoclax^21,22^, a selective BCL2 inhibitor have been approved for CLL treatment. However, evolution and selection of subclones leads to resistance and disease relapse^2^. Most of these new targeted therapies are also disrupting the dialogue of tumor B cells with the microenvironment giving further support to the importance of the microenvironment.

In the present study, we have employed a systems biology approach, based on artificial intelligence and pattern recognition techniques, in order to identify targets and new drugs that can overcome the supportive effect of the tissue microenvironment on CLL cell survival^23^. With this strategy we have created mathematical models that integrate all the available biological, pharmacological, and medical knowledge about CLL to simulate human physiology in silico. Eight of these compounds showed promising results either for their cytotoxicity or viability effects or in many instances for both effects, with minimal changes in their effects when analyzed in coculture. Thus, their action may overturn, at least partially, the supportive effect of the microenvironment. Validation of these 8 selected compounds in primary CLL cells and CLL cell lines in the presence of the stromal HS-5 cell line highlighted that the commercial drug simvastatin (F1) was the most effective and selective out of the 65 compounds tested.

Simvastatin is a commercially approved statin that interferes in the synthesis of cholesterol in the liver by inhibiting the enzyme HMGCR in the mevalonate pathway, thus blocking the synthesis of mevalonate and preventing cholesterol formation^16^. Statins can be classified as natural or fungal-derived (lovastatin, simvastatin, pravastatin), and synthetic (fluvastatin, atorvastatin, rosuvastatin, pitavastatin and cerivastatin). The two groups differ in their ability to inhibit HMGCR and in their lipophilicity^24^. Statins show numerous pleiotropic effects including anti-inflammatory, anti-angiogenic, anti-oxidant and anti-cancer activities^25^. Multiple pleiotropic anti-cancer effects were observed such as induction of cell cycle arrest, apoptosis, inhibition of migration, invasion, metastasis and angiogenesis^26,27^ and particularly, a cytotoxic effect was reported in hematological malignancies^28,29^. Epidemiologic studies suggest improved outcomes in some hematological malignancies in the statins users^30,31^. Particularly in CLL and lymphoma cells, statins induced apoptosis^32,33^. In this way, it has been proposed that upon statin treatment, cellular geranylgeranyl pyrophosphate levels are depleted, resulting in deficient isoprenylation of proteins. This, in turn, would lead to mislocalization and malfunction of such proteins, ultimately causing cell apoptosis^34^. It has also been reported in lymphoma cells that statins induced apoptosis by promoting ROS generation and regulating Akt, Erk and p38 signals via suppression of mevalonate pathway^35^. Furthermore, isoprenoid intermediates of mevalonate pathway might influence the expression of genes participating in the regulation of cell proliferation and transformation^27^. Simvastatin also targets the integrin LFA-1^17^. This protein is a heterodimeric integral membrane protein composed of an alpha chain (LFA-1) and a beta chain (ITGB2) which is expressed on all leukocytes^36^. LFA-1 plays a central role in leukocyte intercellular adhesion through interactions with its receptive counterpart intercellular adhesion molecule 1 (ICAM-1)^37^. LFA-1 is one of the most important adhesion molecules that mediate contact between tumor and stromal cells. Furthermore, cell adhesion has been considered as one of the major causes of primary drug resistance^38^. Our results showed that the statins (simvastatin, lovastatin, fluvastatin and rosuvastatin) exerted a cytotoxic effect, while no effect was observed using LFA-1 inhibitors. Furthermore, all statins tested reduced significantly the proliferation of CLL cells. When we analyzed the effect of statins and LFA-1 inhibitors on ICAM-mediated adhesion and migration of CLL cells triggered by CXCL12 and CXCL13, key chemokines for CLL cell homing to lymphoid tissues^3^, all statins and the LFA-1 inhibitors induced a significant reduction on CLL adhesion/invasion induced by CXCL12 and CXCL13. With these results and the two putative targets of statins, HMGCR and LFA-1, we can hypothesize that statins might participate in the decrease of cell survival and proliferation by inhibiting the pathway of cholesterol synthesis and, on the other hand, they might target cellular adhesion by inhibiting LFA-1 (Fig. 5E). Binding of statins to the LFA1 I-domain induces a conformational change in LFA-1 and inhibits the interaction of LFA-1 with ICAM1, which could contribute to the effects of statins on cell-adhesion, invasion and inflammation^39^. The effect of statins on primary leukemia stem cells has been reported in the context of a supportive microenvironment^40^. Furthermore, the mevalonate pathway inhibition caused by simvastatin abrogates the protective effect of stromal cells in CLL cells^41^ and statins amplify cytokine signaling in CLL cells suggesting that statins might enhance proliferative responses of CLL cells to inflammatory signals^42^. It has also observed a high incidence of hypercholesterolemia in CLL patients supporting that cholesterol-lowering might impact the disease course^43^. Recently, an association between low-potency lipophilic statin (lovastatin and fluvastatin) use and a reduced CLL risk, with a possible dose–response effect has been reported^44^. Our results emphasize on the fact that statins might play a role on the inhibition of the supportive microenvironment in CLL.

Since statins increase the susceptibility of CLL cells to chemotherapy^33^, and the concomitant use of statin and aspirin is associated with improved outcome in CLL patients receiving fludarabine, cyclophosphamide and rituximab (FCR) chemotherapy^45^, we looked for a potential improvement upon the addition of statins to the current CLL treatments. We identified by systems biology-based in silico screening and validated in cell culture that statins can improve the efficacy of ibrutinib or venetoclax. It has been reported that CLL cells express intrinsic resistance to endogenous glucocorticoid receptors (GR) causing an insensitivity to circadian rhythms of plasma cortisol that could impact B cell homeostasis and contribute to the lymphocytosis of CLL. Ibrutinib may help increase the therapeutic activity of glucocorticoids by non-canonical activation of GR^46^, and statins might block ibrutinib-insensitive signals by elevation in plasma cortisol levels^47^. Moreover, preclinical work has shown that statins enhance the ability of venetoclax and navitoclax to kill CLL cells by downregulating glutamine uptake or metabolism as well as its downstream signaling cascades^48^. Furthermore the response to venetoclax was enhanced among statin users in different clinical trials^49^.

In summary, we propose that systems biology approaches combined with pharmacology can be useful to find new drugs for CLL treatment and to predict new and effective combinations. Our results highlight the possibility of repurposing the widely used drugs, such as statins, to improve the efficacy of the currently available targeted therapies in CLL.

## Methods

### Identification of key molecules involved in CLL microenvironment

The ‘CLL microenvironment’ motive was defined through manual curation of the literature to identify proteins (effectors) with a known role in microenvironment effects. An initial search was done including the following terms ("CLL" [TITLE] AND ("MICROENVIROMENT" [TITLE]" or MOLECULAR" [TITLE] OR "PATHOGENESIS" [TITLE] OR "PATHOPHYSIOLOGY" [TITLE])) AND (English [Language] AND Review[ptyp]. An initial set of key processes involved and the proteins with a leading role were identified. When the role of a specific protein was not clear from the review specific articles where retrieved with searches like "CLL" [TITLE] AND microenvironment" [TITLE] ("name protein long" [TITLE/ABSTRACT]) OR "name protein/gene short" [TITLE/ABSTRACT]) AND (English [Language]). This resulted in the selection of 139 effector proteins extracted from 154 articles in PubMed published until 2017. Effector proteins were evaluated in the context of human functional protein network using different network-based mathematical modeling strategies. Models were built on the bases of a human protein functional interaction network, including physical interactions and modulations, signaling, metabolic relationships, and gene expression regulation. Data were obtained from public (KEGG, REACTOME, INTACT, BIOGRID, HPRD, MATRIXDB, MIPS, DIP, MINT) and private databases and manual curation of scientific literature. The final map includes 12,000 proteins and 180,000 links connecting the proteins. A subset of proteins called ‘Key Proteins’ with a high probability of having a large effect on the retrieved molecular description of the microenvironment was identified using two different mathematical modeling strategies based on artificial neural networks (ANN)^11^ and on sampling methods^12^. ANN infers the probability of the existence of a specific relationship between sets of proteins on the bases of the topology of the network and provides a probabilistic score (from 0 to 100%) with an associated p-value that describes the probability of the results being a true positive result. A score > 91% indicates a very strong relationship (p-value < 0.01); a score between 76 and 91% indicates a strong relationship (p-value 0.01–0.05); a score between 40–76% indicates a medium–strong relationship (p-value 0.05–0.25); and a score < 40% indicates a weak relationship (p-value > 0.25). The likelihood of the individual effectors of CLL microenvironment motive of affecting the whole motive has been measured using this approach. Proteins that are closer to a higher number of other proteins in the microenvironment motive have more chances to be a good target. Modeling based on sampling methods infers the most plausible network interactions and information flow linking a set of input proteins. This modeling strategy allows measures of signal propagation through the network. Using this approach, we have evaluated which of the individual effectors of CLL microenvironment, when their action is reverted, had a major impact on the molecular microenvironment description as a whole.

### Identification of compound library for screening and combination therapies

Drug targets where obtained from BindingDB (https://www.bindingdb.org) and DrugBank (https://www.drugbank) databases. The mathematical model strategy based on (ANN)^11^ was used to select compounds affecting the larger number of key proteins related to the microenvironment motive. Compounds were purchased from Mcule Inc. (Palo Alto, CA) (Supplementary Table S1). Additional compounds to validate the targets selected were described in Supplementary Table S2.

### Cell lines and primary cells culture

We used the CLL cell lines HG3 (ACC 765) and MEC-1 (ACC 497) from the German Collection of Microorganisms and Cell Cultures (DSMZ) and the human bone-marrow derived stromal cell line HS-5 (CRL-11882) from the American Type Culture Collection (ATCC). Mycoplasma contamination in cell lines were routinely tested by PCR. Identification of all cell lines was done using the GenePrint 10 system (Promega, Madison, WI, USA). Primary CLL cells and peripheral blood mononuclear cells (PBMCs) from healthy donors were isolated by Ficoll-Paque sedimentation (GE-Healthcare, Munich, Germany), cryopreserved and stored in the Hematopathology collection registered at the Biobank (Hospital Clínic-IDIBAPS; R121004-094). Informed consent was obtained from all patients and donors (IMP CEIC_09052013CAT). Clinical and biological data of each patient are detailed in Supplemental Table S7. The project was conducted following the principles of the Declaration of Helsinki and approved by the Institutional Review Board of our centre, the research Ethic Committee of the Hospital Clínic of Barcelona (HCB/20ls/0708 and HCB/2018/0942).

CLL cell lines, primary CLL cells and PBMCs were cultured in RPMI 1640 complemented with 10% fetal bovine serum (FBS), 2 mM l-glutamine and 50 µg/mL penicillin/streptomycin (Life Technologies, Carlsbad, CA) and grown in a humidified atmosphere at 37 °C with 5% CO_2_. HS-5 cells were cultured in Dulbecco’s modified Eagle’s medium (DMEM, Life Technologies). HS-5 cells (2 × 10^5^ cells/mL) were plated overnight, and once obtained a confluent stroma monolayer, medium was replaced by CLL cells and drugs were added for 48 h. Afterward, CLL cells were collected by carefully rinsing the wells without disturbing the stroma monolayer.

### Analysis of cytotoxicity and cell viability

HG3 and MEC-1 CLL cell lines (200 000 cells/mL) and primary CLL cells (2 × 10^6^ cells/mL) with > 90% tumor B cells, were incubated alone or in coculture with HS-5 cells (50,000 cells/mL) for 48 h with the compounds at doses ranging from 0.1 to 15 µM. Cell cytotoxicity was quantified by double staining with Annexin-V conjugated to fluorescein isothiocyanate (FITC) and propidium iodide (PI) (eBiosciences, San Diego). Normal PBMCs were labeled simultaneously with anti-CD3-FITC, anti-CD19-Phycoeritrin (PE; Becton Dickinson, Franklin Lakes, NJ, USA) antibodies, and Annexin V-Pacific Blue (Life Technologies). Labeled samples were analyzed on an Attune focusing acoustic cytometer (Life Technologies). Cytotoxicity values were represented relative to untreated control. For analysis of cell viability due to an effect on cell proliferation and/or cell death , cells were cultured with the drugs for 48 h and 0.5 mg/mL MTT (3-(4,5-dimethylthiazolyl-2)-2,5-diphenyltetrazolium bromide) reagent (Sigma-Aldrich, St Louis, MO) was added for 2–6 additional hours before spectrophotometric measurement. Each measurement was made in triplicate. Values were represented using untreated control cells as reference.

### In vitro CLL proliferation assay

CLL primary cells (10^7^ cells) were labeled with 0.5 µM carboxyfluoresceinsuccinimidyl ester (CFSE; Life Technologies) as reported previously^50^. Briefly, 10^5^ cells/200 μL were cultured for 6 days in an enriched RPMI-1640 supplemented with 15 ng/mL recombinant human IL-15 (R&D systems, Minneapolis, MN) to sustain survival and with 0.2 μM CpG DNA TLR-9 ligand (ODN-2006; Invivogen, San Diego, CA) to induce cell proliferation^13^. The percentage of divided cells was determined as the percentage of CD19+ (PE)/Annexin-V-(Pacific Blue) cells showing a decrease in CFSE staining on flow cytometry. Fluorescence-minus-one (FMO) was used as a negative control. Data analysis was performed using FlowJow 10.0.7 software (FlowJo, Ashland, OR).

### Chemotaxis assay

Primary CLL cells were washed twice and maintained in serum-starved in FBS-free RPMI during the whole experiment. Three hours after treatment, cells were diluted to 5 × 10^6^ cells/mL with 0.5% bovine serum albumin (BSA; Sigma-Aldrich, Saint Louis, MI) in PBS and 100 µL (5 × 10^5^ cells) were added to the top chamber of a 6.5 mm diameter and 5 μm pore size transwell culture polycarbonate insert (Corning, Corning, NY), previously overnight coated with ICAM (Peprotech, Rocky Hill, NJ), washed twice with PBS, and transferred to wells containing 600 μL of RPMI with 0.5% BSA with or without 200 ng/mL of human recombinant CXCL12 and CXCL13 (Peprotech) per well. After 3 h of incubation, 100 μL from each lower chamber of the transwell plate were collected in triplicate and viable cells gated and counted on a cytometer for 12 s under a constant flow rate of 500 µL/min. Migration was represented as percentage of migrating cells out of total viable cells added to the transwell.

### Cell confluence assay

HG3 cells and HS-5 were plated at 200,000 cells/mL and 50,000 cells/mL, respectively (100 μL per well) into a 96-well flat bottom plate. Cell growth was monitored for 48 h by recording phase images using the IncuCyte ZOOM live cell imaging system (Essen BioScience, Ltd. Royston Hertfordshire; UK) and confluence algorithm.

### Statistical analysis

Statistical data analysis was performed using Prism 6.01 Graphpad software (San Diego, CA). Results are expressed as mean ± SD. Non-parametric Wilcoxon matched-pairs signed rank test was used to compare the median of a set of samples against a hypothetical median. Comparison between two paired groups of samples was evaluated by the nonparametric Wilcoxon matched-pairs signed-rank test. The nonparametric Mann–Whitney test was used to compare two unpaired groups of data. Statistical significance was considered when P-value < 0.05 (*P < 0.05, **P < 0.01, ***P < 0.001). Receiver (or Relative) operating characteristic (ROC) curves were constructed and the area under ROC curves (AUC) was calculated to evaluate sensitivity and specificity of both metabolic rate and cytotoxicity for each compound tested. Cut-off points on the ROC curves with higher AUC and P < 0.05 were used as selection criteria of effective drugs. The best cut off for MTT assay was 50% (Supplemental Figure S1A) and of 20% for annexin analysis (Supplemental Figure S1B).

## Supplementary Information

Supplementary information.
