# Supplementary material for: Systems biology drug screening identifies statins as enhancers of current therapies in chronic lymphocytic leukemia
Source: Sci Rep. 2020 Dec 17;10:22153. doi: 10.1038/s41598-020-78315-0 (PMC7746765; doi:10.1038/s41598-020-78315-0)
Supplement: Supplementary file 1 — Supplementary information. [file 41598_2020_78315_MOESM1_ESM.pdf]

SUPPLEMENTARY INFORMATION

Systems biology drug screening identifies statins as enhancers of current therapies in chronic lymphocytic leukemia

Neus Gimenez, Rupal Tripathi, Ariadna Giró, Laia Rosich, Mònica López-Guerra, Irene Lopez-Oreja, Heribert Playa-Albinyana, Fabian Arenas, José Manuel Mas, Patricia Perez-Galan, Julio Delgado, Elias Campo, Judith Farrés, Dolors Colomer

Table S1_ CLL microenvironment motive

	Protein (Uniprot ID)	Protein (Name)	Protein (Name)	Causative Effect	PMID
1	Q13685	Angio-associated migratory cell protein	AAMP	1	20940416
2	A1L0T0	Acetolactate synthase-like protein	ILVBL	1	20940416
3	P60709	Actin, cytoplasmic 1	ACTB	-1	19278964<br>20687794
4	P51617	Interleukin-1 receptor-associated kinase 1	IRAK1	1	21642962
5	P63261	Actin, cytoplasmic 2	ACTG1	-1	19278964
6	P13612	Integrin alpha-4 // VLA-4 // CD49d	ITGA4	1	21876768<br>20687794<br>22160019
7	Q15848	Adiponectin	ADIPOQ	-1	19960063<br>20454844<br>18818986
8	P20701	Integrin alpha-L	ITGAL // LFA-1	-1	20687794<br>19293181<br>3048446<br>19934331
9	P03950	Angiogenin	ANG	1	16832815<br>15182336<br>15182336
10	P05556	Integrin beta-1 // VLA-4 // CD29	ITGB1	1	21876768<br>21093051<br>20687794<br>22160019
11	O15123	Angiopoietin-2	ANGPT2	1	20671131<br>17928052<br>20382847
12	P05107	Integrin beta-2	ITGB2	-1	20687794<br>19293181<br>3048446
13	P35226	Polycomb complex protein BMI-1	BMI1	1	22130798
14	Q08881	Tyrosine-protein kinase ITK/TS	ITK	1	25730880
15	Q9BXJ1	Complement C1q tumor necrosis factor-related protein 1	C1QTNF1	1	8751471
16	P35968	Vascular endothelial growth factor receptor 2	KDR // VEGFR2	-1	19965686
17	P06681	Complement C2	C2	1	8751471
18	Q9UJU2	Lymphoid enhancer-binding factor 1	LEF1	1	22446006
19	P10643	Complement component C7	C7	1	8751471
20	P41159	Leptin	LEP	1	20454844<br>20643953<br>17080020<br>11460888
21	P29466	Caspase-1	CASP1	1	25815426
22	Q9Y608	Leucine-rich repeat flightless-interacting protein 2	LRRFIP2	1	19265123
23	Q92583	C-C motif chemokine 17	CCL17	1	20883788<br>21709686<br>20883788
24	P08581	Hepatocyte growth factor receptor	MET	1	20809501<br>10979972
25	Q99731	C-C motif chemokine 19	CCL19	1	20883788<br>20687794<br>17082584<br>15184877
26	P14780	Matrix metalloproteinase-9	MMP9	1	21569005<br>20159608<br>19965686<br>15109528
27	P13500	C-C motif chemokine 2	CCL2	1	22397722<br>19074885<br>20981323
28	Q99836	Myeloid differentiation primary response protein MyD88	MYD88	1	19050243<br>22150006
29	O00585	C-C motif chemokine 21	CCL21	1	20883788<br>20687794<br>17082584<br>15184877
30	Q96MN2	NACHT, LRR and PYD domains-containing protein 4	NALP4	1	25686493
31	O00626	C-C motif chemokine 22	CCL22	1	11981828<br>12688308<br>20883788
32	P59047	NACHT, LRR and PYD domains-containing protein 5	NALP5	1	20940416
33	P10147	C-C motif chemokine 3	CCL3	1	20883788
34	P19838	Nuclear factor NF-kappa-B p105 subunit	NFKB1	1	20863894<br>20148715
35	P13236	C-C motif chemokine 4	CCL4	1	20883788
36	O00221	NF-kappa-B inhibitor epsilon	NFKBIE	1	22675518

37	P32246	C-C chemokine receptor type 1	CCR1	1	19383907 20883788 15001469
38	Q9NPP4	NLR family CARD domain-containing protein 4	NLRC4	1	22323829
39	P51679	C-C chemokine receptor type 4	CCR4	1	20883788 21443542
40	Q9Y239	Nucleotide-binding oligomerization domain-containing protein 1	NOD1	1	19036098
41	P51681	C-C chemokine receptor type 5	CCR5	1	19383907 20883788
42	P04085	Platelet-derived growth factor subunit A	PDGFA	1	20606160 16227675
43	P32248	C-C chemokine receptor type 7	CCR7	1	21347514 20883788
44	P01127	Platelet-derived growth factor subunit B	PDGFB	1	20606160 16227675
45	Q86VB7	Scavenger receptor cysteine-rich type 1 protein M130	CD163	1	21699388
46	P16234	Platelet-derived growth factor receptor alpha	PDGFRA	1	20606160 16227675
47	P16671	Platelet glycoprotein 4	CD36	1	10329920
48	P16284	Platelet endothelial cell adhesion molecule	PECAM1 // CD31	1	12673718 19956559 16621959
49	P28907	ADP-ribosyl cyclase 1	CD38	1	20620968 22289918 21765022
50	Q13393	Phospholipase D1	PLD1	1	19934331
51	P25942	Tumor necrosis factor receptor superfamily member 5	CD40	1	[20687794 22160019 22593611 22475052
52	O43157	Plexin-B1	PLXNB1	1	22446006
53	P29965	CD40 ligand // CD154	CD40LG	1	20687794 22160019 22593611 22475052
54	Q13635	Protein patched homolog 1	PTCH1	1	22130798
55	P04233	HLA class II histocompatibility antigen gamma chain	CD74	1	20357260 21417823
56	Q6ISU1	Pre T-cell antigen receptor alpha	PTCRA	-1	20620968 21078912 17984179
57	P39060	Collagen alpha-1(XVIII) chain // Endostatin	COL18A1	-1	17654059 12693719 12857600
58	P63000	Ras-related C3 botulinum toxin substrate 1	RAC1	-1	19934331 20687794 21940819 21474673
59	P78423	Fractalkine	CX3CL1	1	21546901 22457367
60	P61586	Transforming protein RhoA	RHOA	1	22474251 20488224 19934331
61	P49238	CX3C chemokine receptor 1	CX3CR1	1	21546901 15325098 22457367
62	Q15669	Rho-related GTP-binding protein RhoH	RHOH	1	22474251 20687794
63	P48061	Stromal cell-derived factor 1	CXCL12	1	20687794 22160019 19934331 20883788
64	P18827	Syndecan-1	SDC1	1	9470818 18470728
65	O43927	C-X-C motif chemokine 13	CXCL13	1	20883788
66	P14151	L.-selectin	SELL // CD62L	1	11699221 10233382 19654311 1709244 7520409
67	P19875	C-X-C motif chemokine 2	CXCL2	1	22397722 19074885
68	Q92854	Semaphorin-4D	SEMA4D // CD100	1	22446006
69	Q07325	C-X-C motif chemokine 9	CXCL9	1	20981323
70	Q9HC62	Sentrin-specific protease 2	SENP2	1	30431078

71	P49682	C-X-C chemokine receptor type 3	CXCR3	1	20687794 10393705
72	Q8N474	Secreted frizzled-related protein 1	SFRP1	-1	16423993 22672427
73	P61073	C-X-C chemokine receptor type 4	CXCR4	1	22160019 20687794
74	Q96HF1	Secreted frizzled-related protein 2	SFRP2	-1	20495622
75	P32302	C-X-C chemokine receptor type 5	CXCR5	1	20883788
76	Q9UMX1	Suppressor of fused homolog	SUFU	1	19074837
77	O00571	ATP-dependent RNA helicase DDX3X	DDX3X	1	22150006
78	P04435	T-cell receptor beta chain V region CTL-L17	TCRB	-1	20620968 21078912 17984179
79	O76075	DNA fragmentation factor subunit beta	DFFB	1	20940416
80	P01137	Transforming growth factor beta-1	TGFB1	1	9207409 9720719 9159168 15927846
81	O94907	Dickkopf-related protein 1	DKK1	-1	20618428
82	P61812	Transforming growth factor beta-2	TGFB2	1	16785782
83	Q9UBU2	Dickkopf-related protein 2	DKK2	-1	22672427
84	P07996	Thrombospondin-1	THBS1 // TSP1	-1	19604237 18423023 10329920 10545994
85	Q9UBP4	Dickkopf-related protein 3	DKK3	-1	22672427
86	P35590	Tyrosine-protein kinase receptor Tie-1	TIE1	1	16832815 11248324
87	P09038	Fibroblast growth factor-2	FGF2	1	19960063 17241660 11380405 11700386
88	P58753	Toll/interleukin-1 receptor domain-containing adapter protein	TIRAP/MAL	1	23419703
89	P17948	Vascular endothelial growth factor receptor 1	FLT1 // VGFR1	1	11986954
90	Q15399	Toll-like receptor 1	TLR1	1	19036098 19685493 19036098
91	P35916	Vascular endothelial growth factor receptor 2	FLT4 // VEGFR3	1	14687619
92	O60603	Toll-like receptor 2	TLR2	1	22521894 19685493 19036098
93	P02751	Fibronectin	FN1	1	10025901 20501831 11867687
94	O60602	Toll-like receptor 5	TLR5	1	19685493
95	Q9NPG1	Frizzled-3	FZD3	1	22446006
96	Q9Y2C9	Toll-like receptor 6	TLR6	1	19685493 19036098
97	P08151	Zinc finger protein GLI1	GLI1	1	22130798
98	Q9NYK1	Toll-like receptor 7	TLR7	1	19685493
99	P10070	Zinc finger protein GLI2	GLI2	1	19074837
100	Q9NR96	Toll-like receptor 9	TLR9	1	20339095 19685493 18474259 19050243
101	P14317	Hematopoietic lineage cell-specific protein	HCLS1	1	17508001 22333038 20530793
102	P01375	Tumor necrosis factor	TNF	1	21242190 12901966 22144129 21487463
103	P14210	Hepatocyte growth factor	HGF	1	20809501 10979972
104	O14788	Tumor necrosis factor receptor superfamily member 11A	TNFRSF11A // RANK	1	16270354
105	Q16665	Hypoxia-inducible factor 1-alpha	HIF1A	1	18423023 21401803 20018914
106	O14836	Tumor necrosis factor receptor superfamily member 13B	TNFRSF13B // TACI	1	22160019 15860672
107	P05362	Intercellular adhesion molecule 1	ICAM1	1	20687794 15142527
108	Q96RJ3	Tumor necrosis factor receptor superfamily member 13C	TNFRSF13C // BAFFR	1	22160019 20956327 19395025 14504101
109	P22301	Interleukin-10	IL10	1	19956173 21242190
110	Q02223	Tumor necrosis factor receptor superfamily member 17	TNFRSF17 // BCMA	1	22160019 15860672

111	P29460	Interleukin-12 subunit beta	IL12AB	1	15176301 12470418
112	P50591	Tumor necrosis factor ligand superfamily member 10	TNFSF10 // TRAIL	-1	19547714 18160669 16699949 15887227
113	P29459	Interleukin-12 subunit alpha	IL12B	1	15176301 12470418
114	Q9Y6Q6	Tumor necrosis factor ligand superfamily member 11	TNFSF11 // RANKL	1	16270354
115	Q8NAC3	Interleukin-17 receptor C	IL17RC	1	25651714
116	O75888	Tumor necrosis factor ligand superfamily member 13	TNFSF13 // APRIL	1	22160019 21543761 21595749 15860672
117	P01584	Interleukin-1	IL1B	1	18271063 9470818 15074015
118	Q9Y275	Tumor necrosis factor ligand superfamily member 13B	TNFSF13B // BAFF	1	22160019 20956327 19395025 15860672
119	P60568	Interleukin-2	IL2	1	16517754 22623161 20544350 19582829
120	Q9H3D4	Tumor protein 63	TP63	1	20357260 21417823
121	Q9HBE4	Interleukin-21	IL21	-1	17447063
122	Q13077	TNF receptor-associated factor 1	TRAF1	1	12411322 21524353
123	O95760	Interleukin-33	IL33	1	24217710
124	Q12933	TNF receptor-associated factor 2	TRAF2	1	20863894 12411322 21524353
125	P05112	Interleukin-4	IL4	1	20687794 21768328 20716767
126	P19320	Vascular cell adhesion protein 1	VCAM1	1	22160019
127	P05231	Interleukin-6	IL6	1	22475215 21465189 15176301
128	P15692	Vascular endothelial growth factor A	VEGFA	1	19616847 18423023 21519633 21054149
129	P10145	Interleukin-8	IL8 // CXCL8	1	16270354
130	P08670	Vimentin	VIM	1	20620968 6537890 21209908
131	P42768	Wiskott-Aldrich syndrome protein	WAS	-1	20687794 12351399 18223168
132	Q9Y5W5	Wnt inhibitory factor 1	WIF1	-1	18765431 18765431
133	Q9GZT5	Protein Wnt-10a	WNT10A	1	20473358
134	Q9UBV4	Protein Wnt-16	WNT16	1	22446006
135	P56703	Proto-oncogene Wnt-3	WNT3	1	14523464
136	Q9H1J7	Protein Wnt-5b	WNT5B	1	20473358
137	Q9Y6F9	Protein Wnt-6	WNT6	1	20473358
138	O14904	Protein Wnt-9a	WNT9A // WNT14	1	22446006
139	P43403	Tyrosine-protein kinase ZAP-70	ZAP70	1	20687794 18358929 14726163 22151263

List of the 139 effector proteins extracted from 154 articles in PUBMED publsihed until 2017. PMID of the references used to select the protein are listed. The causative effect denotes if it is the protein being more active (1) or more inactive (-1) the cause of the pathological behaviour according to the literature.

.

Table S2: CLL microenvironment Key Proteins

	Uniprot	PROTEIN	GENE	Causative Effect
1	O43566	Regulator of G-protein signaling 14	*RGS14*	1
2	P12931	Proto-oncogene tyrosine-protein kinase Src	*SRC*	-1
3	P25105	Platelet-activating factor receptor	*PTAFR*	-1
4	P61224	Ras-related protein Rap-1b	*RAP1B*	1
5	P62834	Ras-related protein Rap-1A	*RAP1A*	1
6	Q15139	Serine/threonine-protein kinase D1	*PRKD1*	1
7	Q9UL17	T-box transcription factor TBX21	*TBX21*	1
8	P40763	Signal transducer and activator of transcription 3	*STAT3*	-1
9	Q04206	Transcription factor p65	*RELA*	-1
10	P31994	Low affinity immunoglobulin gamma Fc region receptor II-b	*FCGR2B*	1
11	O00206	Toll-like receptor 4	*TLR4*	-1
12	O00585	C-C motif chemokine 21	*CCL21*	-1
13	O14625	C-X-C motif chemokine 11	*CXCL11*	-1
14	O14788	Tumor necrosis factor ligand superfamily member 11	*TNFSF11*	-1
15	O14904	Protein Wnt-9a	*WNT9A*	-1
16	O15123	Angiopoietin-2	*ANGPT2*	-1
17	O60602	Toll-like receptor 5	*TLR5*	-1
18	O60603	Toll-like receptor 2	*TLR2*	-1
19	O75888	Tumor necrosis factor ligand superfamily member 13	*TNFSF13*	-1
20	P01127	Platelet-derived growth factor subunit B	*PDFFB*	-1
21	P01375	Tumor necrosis factor	*TNF*	-1
22	P02778	C-X-C motif chemokine 10	*CXCL10*	-1
23	P04085	Platelet-derived growth factor subunit A	*PDGFA*	-1
24	P05771	Protein kinase C beta type	*PRKCB*	-1
25	P09038	Fibroblast growth factor 2	*FGF2*	-1
26	P10145	Interleukin-8	*CXCL8*	-1
27	P10147	C-C motif chemokine 3	*CCL3*	-1
28	P13236	C-C motif chemokine 4	*CCL4*	-1
29	P13500	C-C motif chemokine 2	*CCL2*	-1
30	P14210	Hepatocyte growth factor	*HGF*	-1

	Uniprot	PROTEIN	GENE	Causative Effect
31	P19838	Nuclear factor NF-kappa-B p105 subunit	*NFKB1*	-1
32	P19875	C-X-C motif chemokine 2	*CXCL2*	-1
33	P32246	C-C chemokine receptor type 1	*CCR1*	-1
34	P48061	Stromal cell-derived factor 1	*CXCL12*	-1
35	P49238	CX3C chemokine receptor 1	*CX3CR1*	-1
36	P78423	Fractalkine	*CX3CL1*	-1
37	Q02223	Tumor necrosis factor receptor superfamily member 17	*TNFRSF17*	-1
38	Q07325	C-X-C motif chemokine 9	*CXCL9*	-1
39	Q08881	Tyrosine-protein kinase ITK/TSK	*ITK*	-1
40	Q15389	Angiopoietin-1	*ANGPT1*	-1
41	Q15399	Toll-like receptor 1	*TLR1*	-1
42	Q15848	Adiponectin	*ADIPOQ*	1
43	Q86VB7	Scavenger receptor cysteine-rich type 1 protein M130	*CD163*	-1
44	Q92583	C-C motif chemokine 17	*CCL17*	-1
45	Q92854	Semaphorin-4D	*SEMA4D*	-1
46	Q99731	C-C motif chemokine 19	*CCL19*	-1
47	Q99836	Myeloid differentiation primary response protein MyD88	*MYD88*	-1
48	Q9BXR5	Toll-like receptor 10	*TLR10*	-1
49	Q9GZT5	Protein Wnt-10a	*WNT10A*	-1
50	Q9H1J7	Protein Wnt-5b	*WNT5B*	-1
51	Q9HB19	Pleckstrin homology domain-containing family A member 2	*PLEKHA2*	-1
52	Q9UBP4	Dickkopf-related protein 3	*DKK3*	1
53	Q9UBU2	Dickkopf-related protein 2	*DKK2*	1
54	Q9Y275	Tumor necrosis factor ligand superfamily member 13B	*TNFSF13B*	-1
55	Q9Y2C9	Toll-like receptor 6	*TLR6*	-1
56	Q9Y608	Leucine-rich repeat flightless-interacting protein 2	*LRRFIP2*	-1
57	Q9Y6F9	Protein Wnt-6	*WNT6*	-1

Causative effect referers to the state of the protein (more active +1, less active -1) that contributes to produce CLL.

Table S3: List of compounds

#	Internal ID	Mcule ID	Systematic name	Common Name
1	A1	MCULE-2510363079	N-(4-methyl-5-{2-[(3-nitrophenyl)amino]-1,3-thiazol-4-yl}-1,3-thiazol-2-yl)acetamide	N.A.
2	A2	MCULE-8191036162	2-(3,4-dihydroxyphenyl)-5-hydroxy-7-{[(2S,3R,4S,5S,6R)-3,4,5-trihydroxy-6-(hydroxymethyl)oxan-2-yl]oxy}-4H-chromen-4-one	N.A.
3	A3	MCULE-8792621521	1-(4-acetylphenyl)-3-{2-[3-(prop-1-en-2-yl)phenyl]propan-2-yl}urea	N.A.
4	A4	MCULE-6000022702	3-{3-[4-(dimethylamino)phenyl]prop-2-enoyl}-4-hydroxy-2H-chromen-2-one	N.A.
5	A5	MCULE-7249710756	1-(4-methylbenzenesulfonyl)-1H-1,3-benzodiazol-2-amine	N.A.
6	A6	MCULE-8365251586	N-{3,5-dimethyl-1-[(2-methylphenyl)methyl]-1H-pyrazol-4-yl}-4H,5H-naphtho[2,1-d][1,2]oxazole-3-carboxamide	N.A.
7	A7	MCULE-3618750171	[(4-carbamoylphenyl)carbamoyl]methyl 3-(2H-1,3-benzodioxol-5-yl)prop-2-enoate	N.A.
8	A8	MCULE-9291497335	3-amino-2-(2-{[5-(2H-1,3-benzodioxol-5-yl)-1,3,4-oxadiazol-2-yl]sulfanyl}acetyl)but-2-enenitrile	N.A.
9	A9	MCULE-9607341619	1-[2,5-dimethyl-1-(prop-2-en-1-yl)-1H-pyrrol-3-yl]-2-[(6-methyl-2-nitropyridin-3-yl)oxy]ethan-1-one	N.A.
10	A10	MCULE-6560812611	3-(4-hydroxyphenyl)-1-phenylprop-2-en-1-one	N.A.
11	A11	MCULE-2266778062	5,5-dimethyl-2-{[(pyridin-3-yl)amino]methylidene}cyclohexane-1,3-dione	N.A.
12	A12	MCULE-9723270257	3-cyano-3-[(2Z)-2,3-dihydro-1H-1,3-benzodiazol-2-ylidene]-2-oxopropyl 2-[(4-bromo-2-methylphenyl)sulfanyl]acetate	N.A.
13	B1	MCULE-7885672138	6,7-dihydroxy-4-({[5-(3,4,5-trimethoxyphenyl)-1,3,4-oxadiazol-2-yl]sulfanyl}methyl)-2H-chromen-2-one	N.A.
14	B2	MCULE-6680323146	N-(2-methylphenyl)-5-[(oxan-2-ylmethyl)sulfanyl]-1,3,4-thiadiazol-2-amine	N.A.
15	B3	MCULE-4044779367	4-(3-methoxypropyl)-4H-1,2,4-triazole-3-thiol	N.A.
16	B4	MCULE-9334292863	2-{4-[5-methyl-2-(propan-2-yl)phenoxymethyl]phenyl}-1,3,4-oxadiazole	N.A.
17	B5	MCULE-1772644300	2-chloro-5H,6H,7H,8H,9H,10H-cyclohepta[b]indole-6-carboxamide	N.A.
18	B6	MCULE-5533294331	(1S,2R,13R,14S,17R,18S)-17-ethynyl-2,18-dimethyl-7-oxa-6-azapentacycloicosa-4(8),5,9-trien-17-ol	Danazol
19	B7	MCULE-3157000542	3-(4-chlorophenyl)-6-(4-fluorophenyl)-5-methylpyrazolo[1,5-a]pyrimidin-7-amine	N.A.
20	B8	MCULE-2559361853	1-[(4-methoxyphenyl)methyl]-2-(2-phenylethenyl)-1H-1,3-benzodiazole	N.A.
21	B9	MCULE-2997420086	N-(3-chloro-4-methylphenyl)-2-[3-chloro-5-(trifluoromethyl)pyridin-2-yl]acetamide	N.A.
22	B10	MCULE-4158159239	ethyl 2-[(propan-2-yl)carbamoyl]-3-{[3-(trifluoromethyl)phenyl]carbamoyl}cyclopropane-1-carboxylate	N.A.
23	B11	MCULE-9091986717	2-phenyl-5-(1H-pyrazol-3-yl)-1,3-thiazole	PHENYL-5-(1H-PYRAZOL-3-YL)-1,3-THIAZOLE
24	B12	MCULE-6640334579	4-methyl-N-(quinoxalin-6-yl)benzamide	N.A.
25	C1	MCULE-5893043131	propanedioic acid	Malonic acid
26	C2	MCULE-2979302446	5,7-dihydroxy-3-(4-methoxyphenyl)-6,8-bis(piperidin-1-ylmethyl)-4H-chromen-4-one	N.A.
27	C3	MCULE-9740144074	2-(3-benzoylphenyl)propanoic acid	Ketoprofen
28	C4	MCULE-5948863568	3,5-diamino-N-carbamimidoyl-6-chloropyrazine-2-carboxamide hydrochloride	Amipramidin Amiloride
29	C5	MCULE-7734253699	5-chloro-7-{[4-(pyridin-2-yl)piperazin-1-yl]methyl}quinolin-8-ol	N.A.
30	C6	MCULE-3879834235	[3,4,5-tris(acetyloxy)-6-sulfanyloxan-2-yl]methyl acetate	Auranofin

31	C7	MCULE-7254287531	2-[4-(dimethylamino)phenyl]-3,6-dimethyl-1,3-benzothiazol-3-ium chloride	N.A.
32	C8	MCULE-5620960445	{[(methylsulfanyl)methanethioyl]amino}(1-phenylethylidene)amine	N.A.
33	C9	MCULE-8575702894	N-(2-phenylphenyl)thiophene-2-carboxamide	N.A.
34	C10	MCULE-8679857296	1-(3-chlorophenyl)-3-[4-(dimethylamino)phenyl]urea	N.A.
35	C11	MCULE-6627172552	N-{10,13-dioxa-4-thia-6-azatricyclo[7.4.0.0?,?]trideca-1,3(7),5,8-tetraen-5-yl}-2-methoxybenzamide	N.A.
36	C12	MCULE-2208162052	2-(4-ethylphenyl)-2-oxoethyl 3-{4-methyl-1,3-dioxo-1H,2H,3H-pyrrolo[3,4-c]quinolin-2-yl}benzoate	N.A.
37	D1	MCULE-7541888215	(4E)-6-(chloromethyl)-4-[(naphthalen-2-yl)imino]-4,5-dihydro-1,3,5-triazin-2-amine	N.A.
38	D2	MCULE-3296821986	2-(4-propoxyphenyl)imidazo[1,2-a]pyridine	N.A.
39	D3	MCULE-5608953652	10-amino-3-azatricyclo[7.3.1.0?,??]trideca-1(13),5,7,9,11-pentaene-2,4-dione	6-AMINO-BENZO[DE]ISOQUINOLINE-1,3-DIONE
40	D4	MCULE-9880228788	N-[(1E)-amino({[(2E)-2,3-dihydro-1,3-benzothiazol-2-ylidene]amino})methylidene]-3-methylbutanamide	N.A.
41	D5	MCULE-7960548249	5-oxo-1H,2H,3H,4H,5H-chromeno[3,4-b]pyridin-4-ium-9-olate	N.A.
42	D6	MCULE-6404224369	N-(5-benzyl-1,3-thiazol-2-yl)thiophene-2-carboxamide	N.A.
43	D7	MCULE-3721465696	4-[3-(4-aminophenoxy)phenoxy]aniline	N.A.
44	D8	MCULE-7823638137	2-(4-tert-butylphenyl)-1,3-benzothiazole	N.A.
45	D9	MCULE-9761380121	1-(2H-1,3-benzodioxol-5-yl)-3-[(2-bromophenyl)amino]prop-2-en-1-one	N.A.
46	D10	MCULE-1144303704	N-(4-fluorophenyl)-5-nitropyridin-2-amine	N.A.
47	D11	MCULE-2502936802	17-{[(3-hydroxyphenyl)methylidene]amino}-17-azapentacyclo[6.6.5.0?,?.0?,??.0??,??]nonadeca-2,4,6,9(14),10,12-hexaene-16,18-dione	N.A.
48	D12	MCULE-5228315948	1-(2,5-dimethylphenyl)-3-[2-(1H-indol-3-yl)ethyl]thiourea	N.A.
49	E1	MCULE-5990662836	N-(6-chloro-1,3-benzothiazol-2-yl)furan-2-carboxamide	N.A.
50	E2	MCULE-3379988083	N-(2-methoxyphenyl)-4-(thiophen-2-yl)-1,3-thiazol-2-amine	N.A.
51	E3	MCULE-2521803878	3-methoxy-N-(4-methylphenyl)benzamide	N.A.
52	E4	MCULE-4381636893	1-(4-phenylbenzoyl)-3-(2,4,6-trimethylphenyl)thiourea	N.A.
53	E5	MCULE-6212734898	5-[(2-phenoxyethyl)sulfanyl]-1-phenyl-1H-1,2,3,4-tetrazole	N.A.
54	E6	MCULE-5694794398	N-[4-(diethylamino)phenyl]-7-(difluoromethyl)-5-methyl-[1,2,4]triazolo[1,5-a]pyrimidine-2-carboxamide	N.A.
55	E7	MCULE-3199876315	4-benzyl-2-[N-(2-hydroxy-5-methylphenyl)carboximidoyl]-6-nitrophenol	N.A.
56	E8	MCULE-1048848056	2-aminoacetamide hydrochloride	Glycinamid
57	E9	MCULE-7690742955	1,3-diethyl-5-[(1-methyl-1H-indol-3-yl)methylidene]-2-sulfanylidene-1,3-diazinane-4,6-dione	N.A.
58	E10	MCULE-3097873461	6-[(4,6-dimethylpyrimidin-2-yl)sulfanyl]-4-methyl-8-oxa-3,5-diazatricyclo[7.4.0.0?,?]trideca-1(13),2(7),3,5,9,11-hexaene	N.A.
59	E11	MCULE-6327754946	2-(2,6-dioxopiperidin-3-yl)-2,3-dihydro-1H-isoindole-1,3-dione	Thalidomide
60	E12	MCULE-5734283547	2-(methylamino)pentanoic acid	N-Methylleucine
61	F1	MCULE-8390617062	(1S,3R,7S,8S,8aR)-8-{2-[(2R,4R)-4-hydroxy-6-oxooxan-2-yl]ethyl}-3,7-dimethyl-1,2,3,7,8,8a-hexahydronaphthalen-1-yl 2,2-dimethylbutanoate	Simvastatin
62	F2	MCULE-3065255051	5-benzoyl-2,3-dihydro-1H-pyrrolizine-1-carboxylic acid	ROX-888
63	F3	MCULE-5698614065	2-[(3R,11S,17S,20S,25aS)-11-(4-carbamimidamidobutyl)-3-carbamoyl-20-(1H-indol-3-ylmethyl)-1,9,12,15,18,21-hexaoxo-docosahydro-1H-pyrrolo[2,1-g]1,2-dithia-5,8,11,14,17,20-hexaazacyclotricosan-17-yl]acetic acid	Eptifibatide
64	F4	MCULE-3466233504	ethyl 2-cyano-4-(1-ethyl-1,2-dihydroquinolin-2-ylidene)but-2-enoate	N.A.
65	F5	MCULE-4334386251	5-[2-(4-hydroxyphenyl)ethenyl]benzene-1,3-diol	SRT501/ resveratrol

Compound shaded in gray correspond to drugs

Table S4: Compounds targeting NOD

Compound	Uniprot AC	UniProt Target	UniProt (SwissProt) Recommended Name of Target Chain	IC50 (nM)
A1	Q07820	MCL1_HUMAN	Induced myeloid leukemia cell differentiation protein Mcl-1	902
	P01375	TNFA_HUMAN	Tumor necrosis factor	1,11E+04
	Q9HC29	NOD2_HUMAN	Nucleotide-binding oligomerization domain-containing protein 2	8,61E+03
	Q9Y239	NOD1_HUMAN	Nucleotide-binding oligomerization domain-containing protein 1	9,40E+03
A12	P01375	TNFA_HUMAN	Tumor necrosis factor	>2.00E+4
	P05186	PPBT_HUMAN	Alkaline phosphatase, tissue-nonspecific isozyme	2,41E+04
	P09923	PPBI_HUMAN	Intestinal-type alkaline phosphatase	1,34E+04
	P10696	PPBN_HUMAN	Alkaline phosphatase, placental-like	2,55E+04
	Q9HC29	NOD2_HUMAN	Nucleotide-binding oligomerization domain-containing protein 2	2,00E+04
	Q9Y239	NOD1_HUMAN	Nucleotide-binding oligomerization domain-containing protein 1	2,00E+04
C5	~~Q13285~~	STF1_HUMAN	Steroidogenic factor 1	6,65E+03
	P01375	TNFA_HUMAN	Tumor necrosis factor	6,23E+03
	P34949	MPI_HUMAN	Mannose-6-phosphate isomerase	5,00E+04
	Q9HC29	NOD2_HUMAN	Nucleotide-binding oligomerization domain-containing protein 2	4,88E+03
	Q9Y239	NOD1_HUMAN	Nucleotide-binding oligomerization domain-containing protein 1	4,58E+03
C7	Q9Y239	NOD1_HUMAN	Nucleotide-binding oligomerization domain-containing protein 1	556
	P01375	TNFA_HUMAN	Tumor necrosis factor	762
	P05067	A4_HUMAN	Amyloid beta A4 protein	116-9,000
	P06276	CHLE_HUMAN	Cholinesterase	3.620
	P22303	ACES_HUMAN	Acetylcholinesterase	1,000-5,000

Table S5: List of compounds used for validation of NOD1 and HMDH/ITGAL targets

Internal ID	Targets	IC50 (nM)	Supplier	Catalog number
BDBM62265	NOD1	32	MoltPort-Vitas-M Laboratory	STK299594
	TNFA	216		
BDBM54356	NOD1	4800	ENAMINE	Z19669458
	TNFA	45,6		
	NOD2	36		
GSK583	RIP2	5	Selleck	S8261
	RIP3	16		
NOD IN 1	NOD1	5740	Medchem express	HY-100691
	NOD2	6450		
NODITINIB	NOD1	560	Selleck	ML130
Lovastatin	HMDH	20-20000	Selleck	S2061
	ITGAL / CD11A / LFA-1A	12900 KD		
Fluvastatin	HMDH	28	Selleck	S1909
Rosuvastatin	HMDH	3--6	Selleck	S2169
Lifitegrast	ITGAL / CD11A / LFA-1A	3	Medchem express	HY-19344
BDBM50199033	ITGAL / CD11A / LFA-1A	18-280	BIONET/Key Organics	BMS-587101 / HE-0001

Table S6: Combination of simastatin with other therapies identified by a systems biology approach

Drug A	Drug B	A Probability	B Probability	Combination Probability
Simvastatin (HMDH)	Sorafenib	75,3	83,5	82,5
	**Ibrutinib**		**73,2**	**81,6**
	Alemtuzumab		85,0	79,4
	Ofatumumab		84,3	76,8
	Rituximab		84,3	76,8
	Alpha,Beta-Methylenea		69,4	76,3
	Epratuzumab		62,6	75,7
	**Venetoclax**		**68,3**	**75,7**
	Bosutinib		70,9	73,7
	Idelalisib		60,2	73,1
	Ponatinib		72,6	72,4
	Pomalidomide		51,1	72,3
	Bortezomib		60,5	72,2
	Nelarabine		75,1	71,7
	Tretinoin		40,7	70,7
	Vincristine		70,7	70,0
	Ixabepilone		63,3	67,2
	Pentostatin		68,7	65,7
	Prednisone		22,3	62,3
	Cladribine		38,9	59,1
	Tositumomab		59,6	54,1
	Gemtuzumab ozogamic		44,5	46,9

Prediction values >78 have an associated P< 0,05, values between 78 and 71 have a P<0.1

Table S7. Clinical and biological features of CLL patients

# Patient	Gender/Age at diagnosis	[a] % Tumor cells[a]	[b] Binet/Rai stage	[c] IGHV	Previous treatment	[d] Cytogenetic alterations
**CLL 01**	M/43	90	B/II	m	Chlorambucil	13Q
**CLL 02**	F/53	96	B/II	um	2CDA, CHOP, Allo-SCT	11Q, TRISOMY 12
**CLL 03**	M/28	91	B/II	um	NO	N
**CLL 04**	M/60	90	B/I	m	NO	TRISOMY12
**CLL 05**	M/58	95	B/II	m	RFCM	13Q, 17P
**CLL 06**	F/61	92		um	RFCM	N
**CLL 07**	M/66	95	C/IV	m	No	13Q
**CLL 08**	F/43	95	A/0	m	No	13Q
**CLL 09**	M/69	98	A/0	m	No	N
**CLL 10**	F/69	98	A/0	m	Chlorambucil	N
**CLL 11**	F/52	92	B/II	m	No	TRISOMY12
**CLL 13**	M/59	95	B/II	m	No	13Q
**CLL 14**	M/67	95	B/II	um	Fludarabine, chlorambucil, ofatumumab	13Q
**CLL 15**	F/77	92	A/0	m	NO	N
**CLL 16**	M/67	95	A/1	m	NO	13Q
**CLL 17**	F/61	49	A/0	m	NO	13Q
**CLL 18**	F/62	96	A/0	m	NO	11Q, TRISOMY 12
**CLL 19**	M/58	86	B/II	um	NO	13Q
**CLL 20**	M/49	96	C/IV	m	NO	13Q
**CLL 21**	F/43	97	B/II	um	FCM	N
**CLL 22**	M/49	82	B/I	um	NO	13Q
**CLL 23**	M/54	94	A/0	m	NO	13Q, 17P
**CLL 24**	F/62	96	A/0	m	NO	13Q

Abbreviations: M, male; F, female; ND, not determined; 2CDA,2-chloro-2'-deoxyadenosine; CHOP: Cyclophosphamide, Doxorubicin, Vincristine, Prednisone ; Allo-SCT: allogenic transplant; FCM, fludarabine, cyclophosphamide, mitroxantrone; RFCM, rituximab plus FCM

[a]Percentage of tumoral cells was quantified by flow cytometry labeling of CD5+/CD19+ cells

[b] According to Rai and Binet's classification: Early (Rai 0, Binet A), intermediate (Rai I/II, Binet B) and advanced (Rai III/IV, Binet C) stage disease.

[c] IGVH gene was sequenced following RT-PCR, and aligned to NCBI IgBlast. m, mutated; um, unmutated. Mutated status was assigned when >2% deviation from germline IGHV sequence was present.

[d]Cytogenetic alterations were assessed by FISH. N, normal

Supplemental Figure S1

A

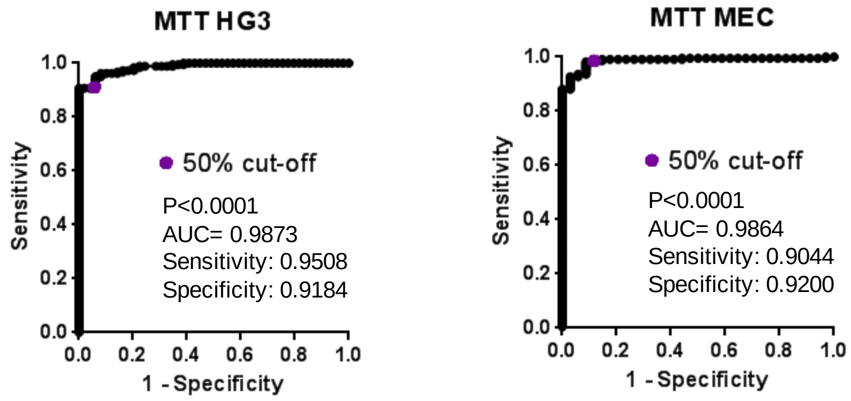

B

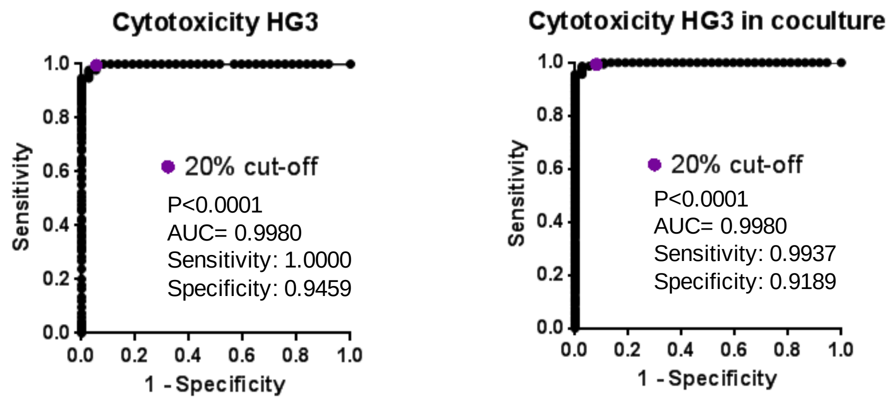

Figure S1. ROC analysis define the threshold to discriminate the effect of the compounds. ROC analysis of the Figure 1 data to define the best discrimination threshold. A) MTT ROC analysis in HG3 and MEC cell lines. B) Cytotoxicity ROC analysis in HG3 alone and in co-culture with HS5 cell line. ****$P < 0.0001$. AUC: Area under the curve.

Supplemental Figure S2

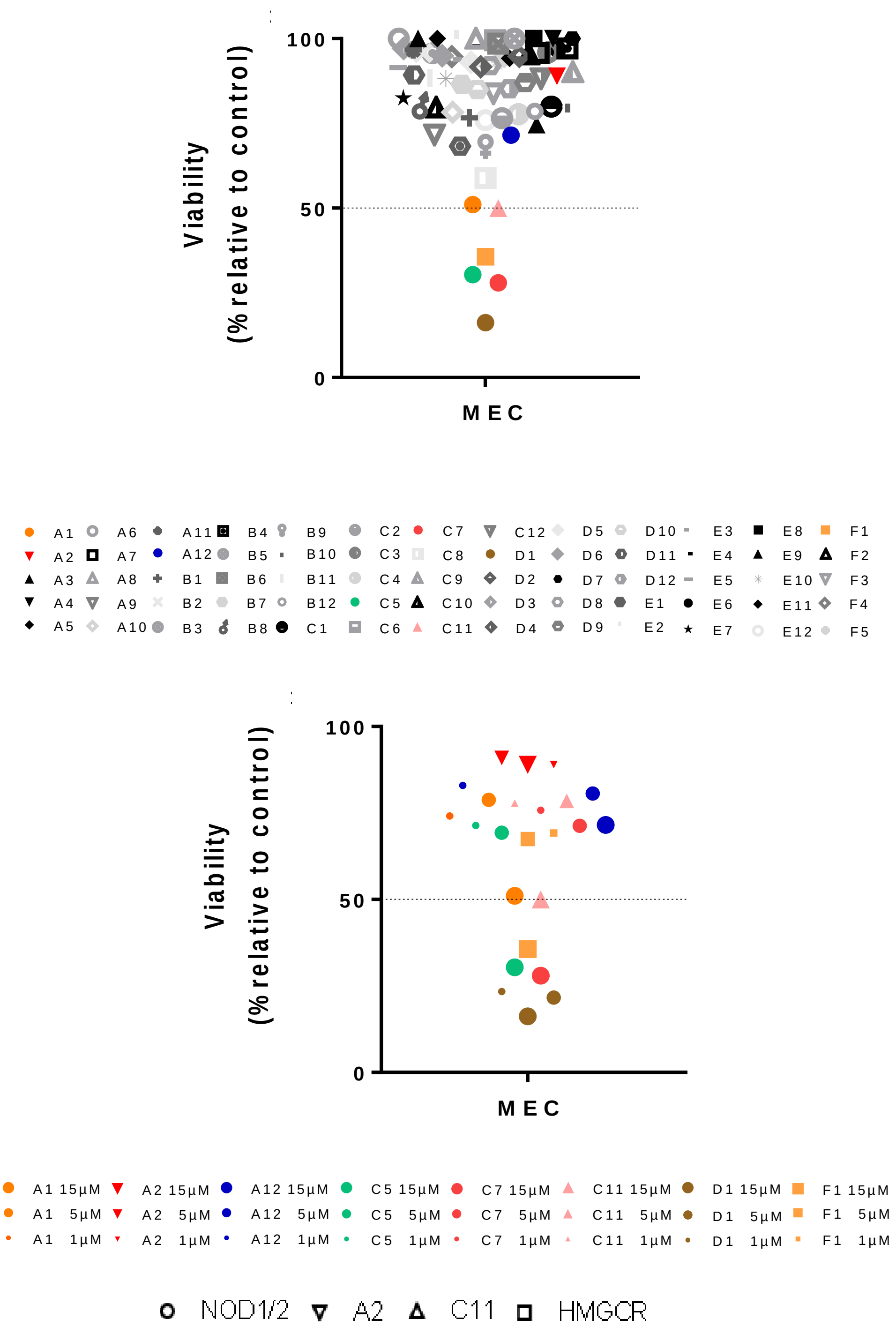

Figure S2. Compound library screening in MEC-1 cell line. Cells were treated for 48 h with the compounds at concentrations from 1 to 15 µM . Each dot represents the mean of 3 independent experiments in different days. Dotted line indicates the threshold to discriminate the effect of the compounds. The cut off for MTT is 50% and for cytotoxicity is 20%. Viability of CLL cells was measured using the MTT assay and is depicted relative to untreated control. A) Viability of cells treated with the compounds at the concentration of 15 µM in MEC-1 cell line. B) Viability of cells treated with the compounds at the concentrations of 1-5-15 µM in the MEC-1 cell line. Round dots: compounds that target NOD1/2; Triangle down-pointing dots: A2; Triangle up-pointing dots: C11; Square dots: compounds that target HMGCR.

Supplemental Figure S3

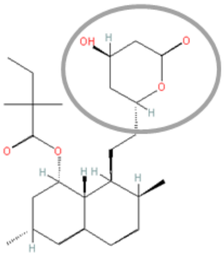

F1	
TARGET	IC_{50} (nM)
HMGCR	11-49
LFA-1	Not detailed

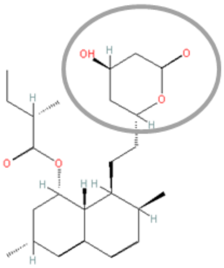

LOVASTATIN	
TARGET	IC_{50} (nM)
HMGCR	20-20100
LFA-1	12900

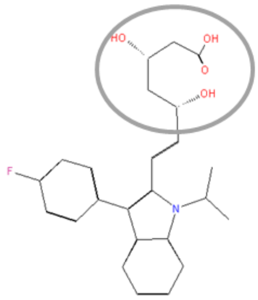

FLUVASTATIN	
TARGET	IC_{50} (nM)
HMGCR	28
LFA-1	NA

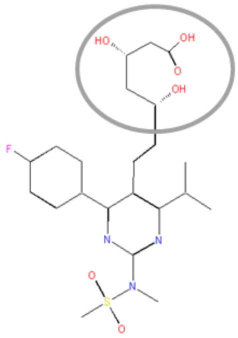

ROSUVASTATIN	
TARGET	IC_{50} (nM)
HMGCR	3.1-5.4
LFA-1	NA

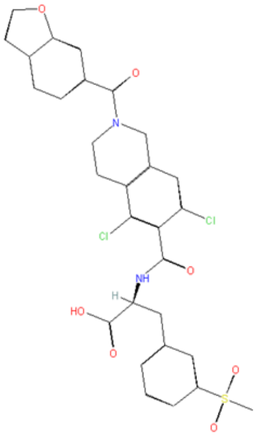

LIFITEGRAST	
TARGET	IC_{50}
LFA-1	9-74

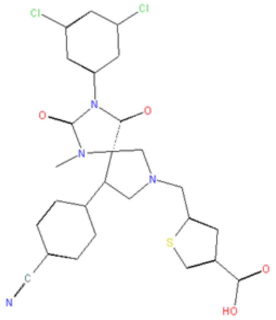

BDBM50199033	
TARGET	IC_{50}
LFA-1	18-280

Figure S3. Chemical structures and IC50 of main targets of statins and LFA-1 inhibitors used.
